# Multi-OMIC profiling of survival and metabolic signaling networks in cells subjected to photodynamic therapy

**DOI:** 10.1007/s00018-016-2401-0

**Published:** 2016-11-01

**Authors:** Ruud Weijer, Séverine Clavier, Esther A. Zaal, Maud M. E. Pijls, Robert T. van Kooten, Klaas Vermaas, René Leen, Aldo Jongejan, Perry D. Moerland, Antoine H. C. van Kampen, André B. P. van Kuilenburg, Celia R. Berkers, Simone Lemeer, Michal Heger

**Affiliations:** 1 Department of Experimental Surgery, Academic Medical Center, University of Amsterdam, Meibergdreef 9, 1105 Amsterdam, AZ The Netherlands; 2 Biomolecular Mass Spectrometry and Proteomics, Bijvoet Center for Biomolecular Research and Utrecht Institute for Pharmaceutical Sciences, Utrecht University, Padualaan 8, 3584 Utrecht, CH The Netherlands; 3 Department of Clinical Chemistry, Laboratory Genetic Metabolic Diseases, Academic Medical Center, University of Amsterdam, Meibergdreef 9, 1105 Amsterdam, AZ The Netherlands; 4 Bioinformatics Laboratory, Academic Medical Center, University of Amsterdam, Meibergdreef 9, 1105 Amsterdam, AZ The Netherlands

**Keywords:** Cancer therapy, Metallated phthalocyanines, Non-resectable perihilar cholangiocarcinoma, Reactive oxygen species, Therapeutic recalcitrance, Tumor targeting

## Abstract

Photodynamic therapy (PDT) is an established palliative treatment for perihilar cholangiocarcinoma that is clinically promising. However, tumors tend to regrow after PDT, which may result from the PDT-induced activation of survival pathways in sublethally afflicted tumor cells. In this study, tumor-comprising cells (i.e., vascular endothelial cells, macrophages, perihilar cholangiocarcinoma cells, and EGFR-overexpressing epidermoid cancer cells) were treated with the photosensitizer zinc phthalocyanine that was encapsulated in cationic liposomes (ZPCLs). The post-PDT survival pathways and metabolism were studied following sublethal (LC_50_) and supralethal (LC_90_) PDT. Sublethal PDT induced survival signaling in perihilar cholangiocarcinoma (SK-ChA-1) cells via mainly HIF-1-, NF-кB-, AP-1-, and heat shock factor (HSF)-mediated pathways. In contrast, supralethal PDT damage was associated with a dampened survival response. PDT-subjected SK-ChA-1 cells downregulated proteins associated with EGFR signaling, particularly at LC_90_. PDT also affected various components of glycolysis and the tricarboxylic acid cycle as well as metabolites involved in redox signaling. In conclusion, sublethal PDT activates multiple pathways in tumor-associated cell types that transcriptionally regulate cell survival, proliferation, energy metabolism, detoxification, inflammation/angiogenesis, and metastasis. Accordingly, tumor cells sublethally afflicted by PDT are a major therapeutic culprit. Our multi-omic analysis further unveiled multiple druggable targets for pharmacological co-intervention.

**Electronic supplementary material:**

The online version of this article (doi:10.1007/s00018-016-2401-0) contains supplementary material, which is available to authorized users.

## Introduction

Photodynamic therapy (PDT) is a non-to-minimally invasive treatment modality for solid cancers that entails the photosensitization of a tumor using light-sensitive compounds called photosensitizers. After the photosensitizer molecules have sufficiently accumulated in the target tissue, the tumor is illuminated with light to activate the photosensitizer molecules [1]. Activated photosensitizers interact with molecular oxygen through energy or electron transfer, leading to the photochemical production of singlet oxygen and superoxide anion, respectively. These reactive oxygen species (ROS) subsequently attack biomolecules in the vicinity of their production site and induce a state of hyperoxidative stress in the illuminated tumor cells in case of an optimal PDT regimen. The oxidative damage in turn results in tumor cell death, microvascular shutdown and corollary tumor hypoxia and hyponutrition, and induction of an anti-tumor immune response (reviewed in [2]), altogether culminating in tumor destruction and removal.

Some types of cancers respond well to PDT and are associated with excellent cure rates, including esophageal carcinoma [3] and basal cell carcinoma [4]. In contrast, the cure rates for nasopharyngeal carcinoma [5] and superficial recurrent urothelial carcinoma HCl [6, 7] are suboptimal with respect to PDT and warrant improvement. Moreover, non-resectable perihilar cholangiocarcinomas respond better to PDT than to any other last-line treatment such as chemotherapy [8], but all available treatments (including PDT) are currently palliative and not curative. The recalcitrant nature of these tumor types to PDT is believed to stem from the use of photosensitizers with suboptimal spectral properties and poor pharmacokinetics as well as the activation of cell survival pathways by tumor cells following PDT [2, 9].

To resolve these issues with a single therapeutic modality, we have developed a 4th-generation photosensitizer-based PDT platform that aims to target pharmacologically relevant locations in the tumor, namely the tumor cells [10], the tumor endothelium [11–13], and the tumor interstitium [14]. The platform employs a 2nd-generation photosensitizer (zinc phthalocyanine, ZnPC) encapsulated in targeted liposomes (making it a 3rd-generation photosensitizer, which was employed in this study) with co-encapsulated molecular inhibitors of survival pathways (making it a 4th-generation photosensitizer) [2, 9–14]. Previously, we demonstrated that PDT of human skin and bile duct cancer cells with liposomal ZnPC and acriflavine, an inhibitor of hypoxia-inducible factor 1α (HIF-1α) [15], increases therapeutic efficacy by downmodulation of HIF-1α-driven survival signaling following PDT [11, 13]. In light of this combined therapy and the broader scope of applicability of the PDT platform technology, it is imperative to map post-PDT survival pathways [9] for every liposomal formulation so as to identify druggable targets beyond those already tested [9]. So far we have mapped PDT-activated survival pathways with respect to the interstitially targeted ZnPC-liposomes [16], but not yet for the endothelium- and tumor cell-targeting liposomes.

Of the three different liposomal formulations that were developed, the most promising is the tumor endothelium-targeting ZnPC formulation. These liposomes, which are cationic and PEGylated, are taken up by cultured endothelial cells [14], macrophages (manuscript in preparation), and tumor cells [11, 13, 14], enabling multi-targeted delivery of the photosensitizer to key locations. Moreover, the liposomes are relatively non-toxic in the absence of light (this study), but become highly toxic to cultured cells upon illumination in the low nanomolar photosensitizer concentration range [11, 13]. Finally, ZnPC distributes to multiple intracellular loci after uptake of the liposomes [17, 18], from which different cell death pathways but also cell survival pathways are activated [2]. In preliminary experiments it was discovered that epidermal growth factor receptor (EGFR), a receptor overexpressed in a multitude of cancers [19] including perihilar cholangiocarcinoma [20, 21], was afflicted by PDT with ZnPC-liposomes. EGFR constitutes an important druggable target in cancer therapy, as evidenced by the approval status of the monoclonal antibodies cetuximab and panitumumab, as well as the kinase inhibitors gefitinib and erlotinib [22].

This study therefore examined the cell survival pathways induced by ZnPC-encapsulating PEGylated cationic liposomes (ZPCLs) in tumor parenchymal and non-parenchymal cell types using a multi-omics approach: transcriptomics, (phospho)proteomics, and metabolomics. The cells that were employed are human umbilical vein endothelial cells (HUVECs) as a model for vascular endothelium; RAW 264.7 murine macrophages as a model for tumor-resident macrophages; human biliary adenocarcinoma (SK-ChA-1) cells as model for PDT-recalcitrant perihilar cholangiocarcinomas; and EGFR-overexpressing human epidermoid carcinoma (A431) cells to further elaborate on the preliminary experimental results. The studies were performed at supralethal light dose (90% lethal concentration, LC_90_), reflective of cells fully affected by PDT, and at sublethal light dose (LC_50_), representative of cells in the distant and peripheral portions of the illuminated tumor, where the fluence rates are insufficient due to light absorption and scattering [23]. Therapeutically, the low-fluence sites are the most important tumor regions because survival signaling is expected to predominate, which may negatively impact therapeutic outcome and facilitate tumor recurrence as has been observed in PDT-treated patients [24].

The most important results of the study were that (1) ZPCLs were not toxic in vitro, which is key for clinical translation, (2) sublethal PDT was associated with extensive survival signaling, which is detrimental to therapeutic outcome, (3) PDT resulted in downregulation of proteins involved in EGFR signaling and cell adhesion, in particular after optimal PDT, and (4) sublethal and optimal PDT both downregulated metabolic pathways involved in energy production, including glycolysis and the tricarboxylic acid (TCA) cycle. The latter two findings are chiefly advantageous for therapeutic efficacy.

## Materials and methods

### Chemicals

1,2-Dipalmitoyl-*sn*-glycero-3-phosphocholine (DPPC) and 3β-[*N*-(*N*′,*N*′-dimethylaminoethane)-carbimoyl]cholesterol (DC-cholesterol) were purchased from Avanti Polar Lipids (Alabaster, AL, USA). β-Mercaptoethanol, cholesterol, chloroform, 1,2-distearoyl-*sn*-glycero-3-phosphoethanolamine-polyethylene glycol (DSPE-PEG, average PEG molecular mass of 2000 amu), ZnPC (97% purity), acetonitrile, 4-(2-hydroxyethyl)-1-piperazineethanesulfonic acid (HEPES), potassium carbonate (K_2_CO_3_), pyridine, sodium chloride (NaCl), sodium deoxycholate, sodium fluoride, sodium orthovanadate, sulforhodamine B (SRB), tris(hydroxymethyl)aminomethane (Tris), and Triton X-100 were obtained from Sigma-Aldrich (St. Louis, MO, USA). Glycerol was purchased from Fisher Scientific (Hampton, NH, USA), and sodium dodecyl sulfate (SDS) and bromophenol blue were obtained from Bio-Rad Laboratories (Hercules, CA, USA). Methanol, perchloric acid (O_4_), and sodium hydroxide (NaOH) were from Merck (Darmstadt, Germany).

All lipids were dissolved in chloroform and stored under a nitrogen atmosphere at −20 °C. ZnPC was dissolved in pyridine at a 178-µM concentration and stored under nitrogen at room temperature (RT) in the dark.

### Cell culture

Human epidermoid carcinoma (A431) cells and murine macrophages (RAW 264.7) were cultured in Dulbecco’s modified Eagle’s medium (DMEM, Lonza, Walkersville, MD, USA) supplemented with 10% fetal bovine serum (FBS) (Bodinco, Alkmaar, the Netherlands), 100 U/mL penicillin, 100 µg/mL streptomycin, and 2 mM l-glutamine (all from Lonza). Human umbilical vein endothelial cells (HUVECs) were isolated as described in [25] and maintained in EndoGro-LS complete culture medium (Merck Millipore, Billerica, MA, USA). HUVECs were grown in Primaria cell culture flasks (Corning Life Sciences, Tewksbury, MA, USA). Human perihilar cholangiocarcinoma (SK-ChA-1) cells were cultured in Roswell Park Memorial Institute (RPMI) 1640 culture medium (Lonza) supplemented with 10% FBS, 100 U/mL penicillin, 100 µg/mL streptomycin, 2 mM l-glutamine, and 143 µM β-mercaptoethanol. All cells were maintained at standard culture conditions (37 °C, 5% CO_2_, 95% air, humidified atmosphere).

### Preparation of ZPCLs

ZPCLs were composed of DPPC, DC-cholesterol, cholesterol, and DSPE-PEG (66:25:5:4, molar ratio) and prepared by the lipid film hydration technique as described previously [13, 16]. Physiological buffer composed of 10 mM HEPES, 0.88% (w/v) NaCl, pH = 7.4, 0.293 osmol/kg [14] was used as hydration solution. ZnPC was incorporated in the liposomal formulation at a ZnPC:lipid molar ratio of 0.003. Liposomal formulations were purged with nitrogen gas and stored at 4 °C in the dark. Under these conditions the liposomal ZnPC remains stable for at least 56 days [13].

### PDT protocol

Cells were seeded in either 6-well (2 mL per well) or 24-well (0.5 mL medium per well) culture plates (Corning Life Sciences) as specified in the corresponding subsections and grown under standard culture conditions. HUVEC, RAW 264.7, SK-ChA-1, and A431 cells were seeded at a density of 0.5  ×  10^5^ cells/mL, 0.5  ×  10^6^ cells/mL, 0.25  ×  10^6^ cells/mL, and 0.5  ×  10^6^ cells/mL, respectively, and cultured until confluence in 24 h (48 h for SK-ChA-1 cells). HUVECs were cultured in Primaria culture plates (Corning Life Sciences) throughout the study. After reaching confluence, cells were washed with PBS and incubated with ZPCLs in serum-free supplemented phenol red-free medium for 1 h (drug-light interval) at 37 °C under standard culture conditions. Control cells received an equal volume of physiological buffer. The concentrations of ZPCLs that were used for the different cell types are specified in Table S1. Next, cells were washed with PBS and fresh fully supplemented phenol red-free medium was added. Cells were either returned to the incubator (control and dark toxicity) or irradiated with a 671-nm diode laser (CNI, Changchun, China) at a laser power of 500 mW with a fluence of 15 J/cm^2^. The spot size was set to the exact dimensions of the well (6-wells plate: 9.5 cm^2^, 24-wells plate: 1.9 cm^2^). During the application of PDT, cells were maintained at 37 °C using a hotplate (Cat. No. 97042-616, VWR, Radnor, PA, USA).

### Cell metabolic activity and viability assays

Cell metabolic activity was assessed using the water-soluble tetrazolium salt (WST-1) reagent (Roche Diagnostics, Basel, Switzerland). Cells were seeded in 24-wells plates and cultured until confluence. After a predetermined time interval following PDT, the culture medium was removed and 300 µL of WST-1-containing serum-free and phenol red-free medium (at a 1:25 volume ratio) was added to the wells. After 30 min of incubation under standard culture conditions, the absorbance was read at 450 nm using 600 nm as a reference wavelength (BioTek Synergy HT multi-well plate reader, Winooski, VT, USA). Data were normalized to the average value of the control cells that was set at a metabolic activity of 100%.

After the measurement, the wells were washed with PBS and the protein content was determined with the SRB total protein assay as described by Vichai et al. [26]. SRB absorbance was read at 564 nm using 690 nm as a reference wavelength (BioTek Synergy HT). Data were normalized to the average value of the control cells that was set at a viability of 100%.

### Whole genome expression analysis

Cells were seeded in 6-wells plates and cultured until confluence. Cells were treated using the PDT protocol as described in “PDT protocol” (*n* = 3 per group). Total cellular RNA was extracted using 1 mL of TRIzol (Life Technologies, Carlsbad, CA, USA) according to the manufacturer’s protocol. RNA samples were purified using the NucleoSpin RNA kit (Machery-Nagel, Düren, Germany) and eluted in 30 µL RNAse-free water. The quality control, RNA labeling, hybridization, and data extraction were performed at ServiceXS (Leiden, the Netherlands). The procedure can be found in [16]. Samples for human cell lines were randomly assigned to three Human-HT12 v4 arrays. For the RAW 264.7 cell line, MouseWG-6 v2 arrays were used with control and vehicle samples on one chip and LC_50_ and LC_90_ samples on a second chip.

### Microarray data preprocessing and analysis

Microarray data preprocessing and analysis were performed as described previously [16]. In short, each cell line was analyzed separately with Bioconductor packages (version 2.13) using the statistical software package R (version 3.1.0). Normalization was performed starting from the Illumina sample and control probe profiles by a normexp-by-control background correction, quantile normalization, and log_2_ transformation (limma package). Probes with a detection *P* value of >0.05 (non-expressed) on all arrays for the cell line under study were filtered out. Differential expression between the experimental conditions was assessed with a moderated *t* test using the linear model framework (limma package). Resulting *P* values were corrected for multiple testing using the Benjamini-Hochberg false discovery rate. Corrected *P* values ≤0.05 were considered statistically significant. Probes were reannotated using the Bioconductor IlluminaHumanv4.db and lluminaMousev2.db packages. The microarray data have been deposited in NCBI Gene Expression Omnibus in a MIAME compliant format and are accessible under GEO series accession number GSE84758. Microarray data were confirmed using quantitative reverse transcription polymerase chain reaction (qRT-PCR) since the qRT-PCR data were in agreement with the microarray data (Fig. S1). This also strongly suggests that, for the RAW 264.7 cells, potentially confounding effects due to systematic differences between chips and biological effects of interest (comparison of LC_50_/LC_90_ versus control/vehicle) are limited. In addition, a ROAST gene set test [27] was performed on the downstream targets of each survival pathway (Table S2) to statistically determine whether a survival pathway was either upregulated or downregulated using 10,000 rotations with Benjamini-Hochberg-based multiple testing correction of the mid *P* values.

### qRT-PCR

RNA was extracted as described in “Whole genome expression analysis”. cDNA synthesis and qRT-PCR reactions were performed as described previously [16]. Primer sequences can be found in Table S3. The quantitative analysis of the qRT-PCR data was performed according to Ruijter et al. [28] to calculate the starting concentration (N_0_) of each cDNA template. Gene expression levels were normalized to the expression level of the reference gene ribosomal protein S18 (*RPS18*). Log_2_ fold-changes of the target genes were calculated based on the mean values of the control group.

### Proteomics

#### Harvesting

SK-ChA-1 cells were seeded in 6-wells plates and cultured until confluence. Cells were treated using the PDT protocol as described in “PDT protocol” (*n* = 12 per group). Ninety minutes post-PDT, cells were washed three times with 2 mL PBS and 150 µL of lysis buffer [8 M urea, 0.5% sodium deoxycholate, 50 mM NH_4_HCO_3_, supplemented with cOmplete Mini protease inhibitor cocktail and phosSTOP (both from Roche)] was added to each well that was ensued by 30-min incubation on ice. Lysates were scraped, collected, pooled (to yield *n* = 4 per treatment group), and centrifuged for 15 min at 20,000×*g*. The supernatant was stored at −80 °C for further analysis. Protein concentrations were determined with the bicinchoninic acid (BCA) assay (Thermo Fisher Scientific, Waltham, MA, USA).

#### Affinity purification and digestion

For each sample, 400 µg of proteins was reduced by incubating with 2 µL of 1 M DTT at 56 °C for 25 min, alkylated by adding 4 µL of 200 mM IAA for 30 min at RT in the dark, and digested by Lys-C (enzyme:protein ratio of 1:75) for 4 h at 37 °C. Samples were then diluted four times with 50 mM NH_4_HCO_3_ and digested overnight at 37 °C with trypsin (enzyme:protein ratio of 3:100). Next, 100 µL of acetic acid was added to each sample to precipitate sodium deoxycholate, after which the samples were centrifuged for 15 min at 20,000×*g*. The obtained digests were desalted using 1 cc Sep-Pak C18 cartridges. Phosphoenrichment was performed with Ti-IMAC microcolumns with 250 µg of digests following the protocol previously described in detail [29], while the rest of the digests was kept for proteome analysis.

#### NanoLC–MS/MS analysis

Phosphoproteome and proteome were analyzed by NanoLC-MS/MS using an Agilent 1100 HPLC system (Agilent Technologies, Santa Clara, CA, USA) coupled to a Q Exactive Plus Orbitrap (Thermo Scientific) mass spectrometer. Peptides were trapped at 5 µL/min in 100% solvent A (0.1 M acetic acid in water) on an in-house packed 20 mm × 100 µm ID trapping column (ReproSil-Pur C18-AQ, 3 µm, Dr. Maisch, Ammerbuch, Germany) and then transferred to an in-house packed 50-cm × 50-µm ID analytical column (Poroshell 120 EC-C18, 2.7 μm, Agilent Technologies) maintained at 40 °C. The gradient used for proteome analysis ranged from 10% to 40% solvent B [0.1 M acetic acid in 8:2 (v/v) acetonitrile/water] in 180 min at ~100 nL/min, whereas the gradient for phosphopeptides ranged from 4% to 40% in 120 min. The eluent was sprayed via distal coated emitter tips (New Objective, Woburn, MA, USA) connected to the analytical column. The Q Exactive Plus was operated in data-dependent mode, automatically switching between MS and MS/MS. Full-scan MS spectra (from *m*/*z* 350 to 1500) were acquired in the Orbitrap with a resolution of 60,000 at *m*/*z* 400 (after accumulation to a target value of 500,000). The 20 most intense ions at a threshold above *m/z* 500 were successively selected and fragmented in HCD cells at normalized collision energy of 35% after accumulation to a target value of 10,000.

#### Protein quantification and identification

Data analysis was performed using MaxQuant (version 1.5.2.8) [30] and the integrated search engine Andromeda [31]. For peptide and protein identification, raw files were searched against the human Swissprot database (20,201 entries) with carbamidomethylated cysteine as fixed modification and phosphorylation of serine, threonine, and tyrosine and oxidation of methionine as variable modifications. Trypsin/P was set as the proteolytic enzyme for which up to two missed cleavage sites were allowed. Precursor tolerance was set to 4.5 ppm and fragment ion tolerance to 0.05 Da. Peptide identifications required a minimal length of 7 amino acids and all data sets were adjusted to 1% PSM FDR. For label-free quantification (LFQ), match between runs was selected with a maximum shift time window of 3 min and the intensities of razor and unique peptides were summed up. Resulting protein intensities were then normalized to obtain LFQ intensities. To facilitate further data analysis, the results were imported into Perseus (version 1.5.2.4). Replicates were grouped per condition, and proteins or phosphopeptides identified in less than 3 out of 4 replicates were discarded. A two-tailed t-test was used to assess statistical significance. Phosphopeptide and protein *P* values were corrected by permutation-based FDR correction (FDR 5%). Phosphopeptides were filtered for a localization probability of >0.75 (class 1 sites). Regulated proteins were analyzed using Reactome within the Cytoscape environment and regulated phosphorylation sites were analyzed by Phosphopath [32] within Cytoscape. The mass spectrometry proteomics data have been deposited to the ProteomeXchange Consortium via the PRIDE partner repository with the dataset identifier PXD004320.

### Western blotting

Western Blotting was performed to validate the (phospho)proteomic data (Fig. S2). For these purposes, SK-ChA-1 cells were seeded in 6-wells plates, cultured until confluence, and treated by PDT as described in “PDT protocol” (*n* = 3 per group). Ninety minutes after PDT, cells were washed twice with ice-cold PBS, placed on ice, and lysed in ice-cold RIPA buffer (50 mM Tris, 150 mM NaCl, 1% Triton X-100, 0.5% sodium deoxycholate, 1% SDS) supplemented with cOmplete Mini protease inhibitor cocktail, 10 mM sodium fluoride, and 1 mM sodium orthovanadate. The samples were centrifuged for 15 min at 14,000×*g* (4 °C) and the supernatant was stored for further analysis. Protein lysates were mixed with 4 × SDS sample buffer (200 mM Tris (pH = 6.8), 8% SDS, 40% glycerol, 0.02% bromophenol blue) and boiled for 5 min at 95 °C. Next, samples (20–30 µg) were loaded on a TGX 10% precast gel (Bio-Rad Laboratories) and electrophoresis was performed at 150 V. The gels were blotted onto Amersham Hybond P 0.45 PVDF membranes (GE Healthcare, Little Chalfont, UK) for 2 h at 250 mA at 4 °C. The membranes were blocked for 1 h with 5% BSA (Sigma-Aldrich) in 0.1% Tween 20 Tris-buffered saline (TBST, 20 mM Tris, 150 mM NaCl, pH = 7.6), after which the membranes were incubated overnight with the primary antibody at 4 °C on a rocker. The primary antibodies used were (dilution factor, catalogue number, company): EGFR [1:1000, #4267, Cell Signaling (Danvers, MA, USA)], phospho-ERK (1:1000, #4370, Cell Signaling), phospho-p38 MAPK (1:500, #9216, Cell Signaling), p38 MAPK (1:1000, #9228, Cell Signaling), COX IV (1:1000, #4844, Cell Signaling), and ERK [1:1000, sc-2711270, Santa Cruz Biotechnology (Dallas, TX, USA)]. All primary antibodies were diluted with 5% BSA in TBST. Next, the membranes were washed three times in TBST and incubated with an HRP-conjugated secondary antibody [1:2000, Dako Cytomation (Glostrup, Denmark)] for 1 h at RT. Subsequently, membranes were washed three times with TBST. The enhanced chemiluminescence (ECL) kit (Thermo Scientific) was used as substrate and protein bands were visualized on an ImageQuant LAS 4000 luminometer (GE Healthcare).

### Metabolomics

SK-ChA-1 cells were seeded in 6-wells plates and cultured until confluence. Cells were treated using the PDT protocol as described in “PDT protocol” (*n* = 3 per group). After 90 min, the cells were washed with 1 mL cold PBS and the cells were lysed in 1 mL lysis buffer (40% acetonitrile, 40% methanol, 20% water). The cells were scraped and transferred to 2-mL centrifuge tubes that were shaken for 10 min at 4 °C. Next, the samples were centrifuged for 15 min at 20,000×*g* (4 °C), after which the supernatant was aspirated and stored at −80 °C. LC-MS analysis was performed on an Exactive mass spectrometer (Thermo Scientific) coupled to a Dionex Ultimate 3000 autosampler and pump (Thermo Scientific). The MS operated in polarity-switching mode with spray voltages of 4.5  and −3.5  kV. Metabolites were separated using a Sequant ZIC-pHILIC column [2.1 × 150 mm, 5 µm, guard column 2.1 × 20 mm, 5 µm (Merck)] using a linear gradient of acetonitrile and eluent A (20 mM (NH_4_)_2_CO_3_, 0.1% NH_4_OH in ULC/MS grade water [Biosolve, Valkenswaard, the Netherlands)]. The flow rate was set to 150 µL/min. Metabolites were identified and quantified using LCquan software (Thermo Scientific) on the basis of exact mass within 5 ppm and further validated in accordance with the retention times of standards. Peak intensities were normalized based on total ion count.

### Nucleotide profiles

SK-ChA-1 cells were seeded in 6-wells plates and cultured until confluence. Cells were treated using the PDT protocol as described in “PDT protocol” (*n* = 3 per group). After 90 min, the cells were washed twice with PBS, placed on ice, and nucleotides were extracted using 200 µL of ice-cold 0.4 M HClO_4_. After 10-min incubation on ice, the samples were centrifuged for 5 min at 10,000×*g* (4 °C) and the nucleotide-containing supernatant was neutralized using 7.5 µL of 5 M K_2_CO_3_. The wells were washed twice with 150 µL 0.2 M NaOH to remove residual proteins, which was added to the protein-containing dry pellet as obtained in the previous centrifugation step. In addition, 300 µL of 0.8 M HClO_4_ was added to the protein fraction. After mixing thoroughly, the samples were centrifuged for 5 min at 10,000×*g* (4 °C) and the protein-containing pellet was dissolved in 200 µL of 0.2 M NaOH. Protein content was determined using the bicinchoninic acid assay protein kit (Thermo Scientific).

Nucleotide extracts were analyzed by high-performance liquid chromatography (HPLC) using a Partisphere 5-μm SAX cartridge column (Cat. No. 4621-0505, Hichrom, Reading, United Kingdom). Nucleotides were eluted with a gradient from 100% buffer A (100-fold dilution of buffer B) to 70% buffer B (0.75 M NaH_2_PO_4_^−^, pH = 4.55) in 50 min at a flow rate of 1 mL/min.

### Statistical analysis

**Fig. 1:**
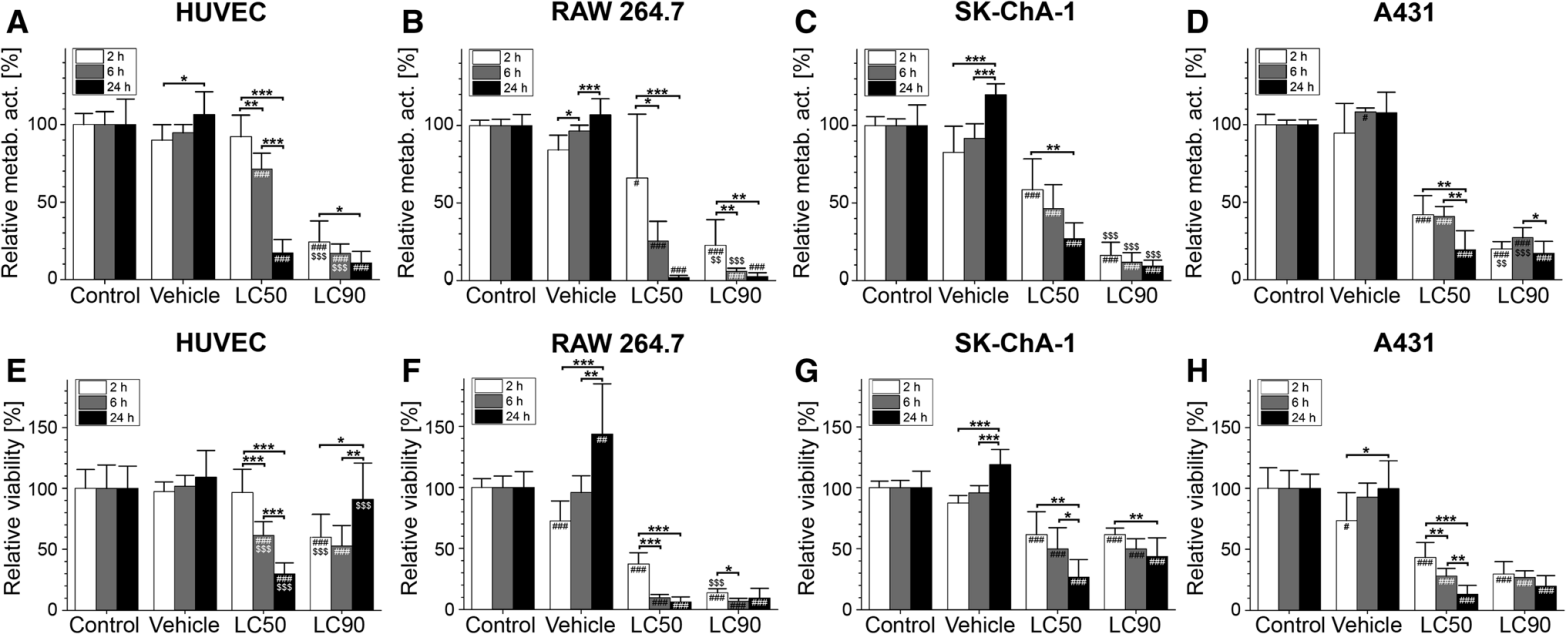
Cell viability after ZPCL-PDT. HUVEC, RAW 264.7, SK-ChA-1, and A431 cells were incubated with ZPCLs (concentrations can be found in Table S1) and treated with PDT. Two hours (*white bar*), 6 h (*light gray bar*), and 24 h (*dark gray bar*) after PDT, cell viability was determined using the **a**–**d** WST-1 and **e**–**h** SRB assay (*n* = 8 per group). Readers are referred to the experimental section for the significance of the statistical symbols. Metab. act., metabolic activity

Statistical analysis was performed in GraphPad Prism 6 (GraphPad Software, La Jolla, CA, USA). Normality was tested with the D’Agostino Pearson omnibus test. Differences between normally distributed variables were analyzed with a one-way ANOVA with Bonferroni post hoc test. Intergroup differences were indicated with (*) and differences between the treated groups and the control group at the same time point were indicated with (#). Differences between a condition and the previous condition at the same time point are, when relevant, indicated with ($) (pertains only to Fig. 1). A single, double, and triple sign indicate a *P* value of  ≤ 0.05, ≤0.01, and  ≤ 0.001, respectively. Data are presented as mean  ±  SD throughout the manuscript.

## Results

### PDT induces photosensitizer concentration- and time-dependent cell death

To correlate the transcriptomic-, (phospho)proteomic-, and metabolomic responses to the extent of PDT-induced cell death, the viability of HUVEC, RAW 264.7, SK-ChA-1, and A431 cells was determined first as a function of time after PDT at previously calculated LC_50_ and LC_90_ concentrations (details can be found in Table S1). The effect of PDT on cells was assessed with the WST-1 and SRB assays. WST-1 is a measure of mitochondrial metabolic activity [33] and therefore represents a parameter of early onset cell demise. In contrast, SRB stains total protein and is therefore used as a parameter of late, fully executed cell death.

The ZPCLs exhibited no deleterious effect on metabolic activity (Fig. 1a–d) or cell viability (Fig. 1e–h) in any of the cell types in the absence of laser irradiation, indicating that the ZPCLs imparted no dark toxicity. The loss of metabolic activity (Fig. 1a–d) and extent of cell death (Fig. 1e–h) were more pronounced in the LC_90_ group versus the LC_50_ group and occurred in a time-dependent manner. The loss of metabolic activity is in line with the localization of ZnPC to mitochondrial membranes [2] and the post-PDT induction of mitochondrial permeability transition [12]. Typically, cells were most afflicted at the longest incubation time, underscoring that metabolic perturbations and execution of cell death pathways are progressive during at least 24 h after PDT. Unexpectedly, the LC_90_ HUVECs showed significantly higher cell viability 24 h after PDT compared to 2 h post-PDT (Fig. 1e). HUVECs in the LC_90_ group were also more resilient to treatment 24 h following PDT than HUVECs in the LC_50_ group (Fig. 1e).

### PDT at LC_90_ has greater transcriptional effects than at LC_50_, but the effect size is cell type-dependent

**Fig. 2:**
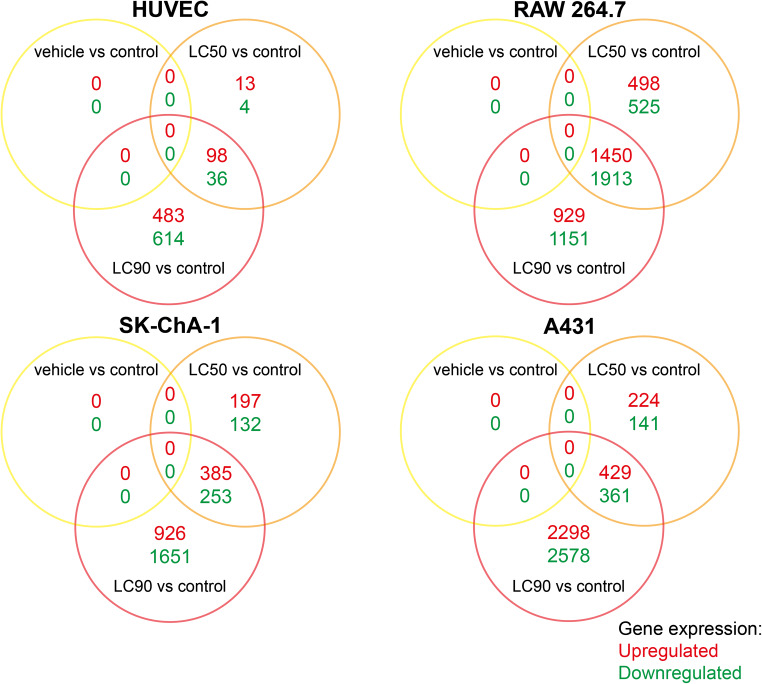
Gross transcriptional response 90 min after ZPCL-PDT. The Venn diagrams show the number of upregulated (*red*) and downregulated (*green*) genes compared to the control group (FDR < 0.05), as well as the overlapping genes between the vehicle (dark toxicity), LC_50_, and LC_90_ groups (*n* = 3 per group). The total number of upregulated and downregulated genes per PDT regimen (*full circle*) equals the sum of all values enveloped by the respective *circle*

To gain insight in the early transcriptomic response after PDT with ZPCLs, non-illuminated and PDT-treated cells were harvested 90 min after (control) treatment and the transcriptome was analyzed by whole genome microarray, summarized in Fig. 2, and correlated to cell viability. This toxicogenomics approach corroborated the absence of dark toxicity of ZPCLs (Fig. 2, vehicle vs control), given that of all screened genes, none were dysregulated compared to control. The same had been observed previously with the ZnPC-encapsulating interstitially targeted liposomes [16], which differ from the ZPCLs in that they lack DC-cholesterol in the membrane and therefore bear a neutral surface charge rather than a cationic charge.

In case of the interstitially targeted liposomes, the milder PDT protocol (irradiation of cells at 50 mW) induced more profound transcriptional dysregulation than the severe PDT regimen (500 mW laser irradiation) [16]. In contrast, the extent of mRNA dysregulation following PDT with ZPCLs was most pronounced in the LC_90_ groups compared to the LC_50_ groups (Fig. 2). The overlap between genes dysregulated in both the LC_90_ and LC_50_ groups was also cell type-specific. The highest number of commonly afflicted genes was observed in RAW 264.7 cells (3363), followed by A431 (790), SK-ChA-1 (638), and HUVEC (134) cells.

### PDT-mediated induction of survival signaling

**Fig. 3:**
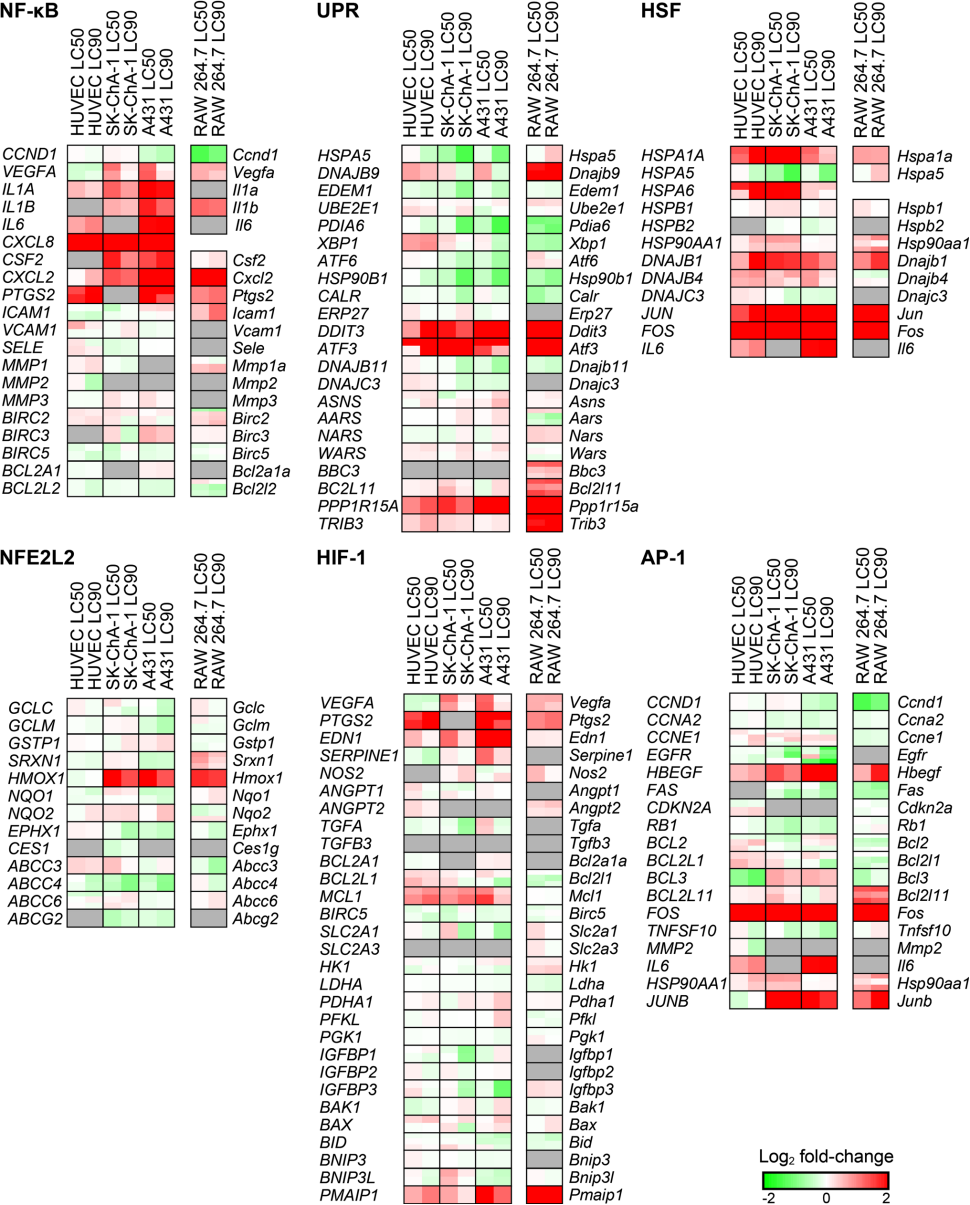
Transcriptional response following ZPCL-PDT. Expression analysis of genes that are involved in NF-кB, UPR, HSF, NFE2L2, HIF-1, and AP-1 signaling as shown by the log_2_ fold-change (*lower right corner*). All comparisons were made between the PDT-treated groups versus the control group (*n* = 3 per group). A gene may correspond to multiple probes as indicated by horizontal splits. Each gene is divided in two halves corresponding to the LC_50_ (*left*) and LC_90_ (*right*) group. *Gray boxes* signify probes that exhibited poor quality or were not included in the gene expression analysis

The basis of therapeutic recalcitrance towards PDT may partly originate from the induction of survival signaling after PDT [16]. PDT activates six major pathways that encompass a nuclear factor of kappa light polypeptide gene enhancer in B cells (NF-кB)-mediated inflammatory response, a proteotoxic stress response via the unfolded protein response (UPR) and heat shock transcription factor (HSF)-mediated response, an activator protein 1 (AP-1)-mediated immediate early gene response, a HIF-1-mediated hypoxia-induced stress response, and a nuclear factor (erythroid-derived 2)-like (NFE2L2)-mediated antioxidant response [9]. The pathways have been described in detail in [16, 34]. The microarray expression data were superimposed on these pathways [Fig. S3 (with pathways and transcriptional targets) and Fig. 3 (transcriptional targets only)].

The downstream targets of the survival pathways were analyzed with a ROAST gene test to determine whether a pathway was differentially regulated in response to PDT. All cell types upregulated the NF-кB, AP-1, and HSF survival pathways at LC_50_ and LC_90_, whereas only the LC_50_ groups exhibited upregulation of HIF-1-mediated signaling (Table S4). Importantly, HIF-1-, UPR-, AP-1-, and NF-кB-associated genes were less extensively dysregulated in the LC_90_ group of the tumor-derived cell lines (A431, SK-ChA-1) compared to the LC_50_ group. In contrast, the LC_90_ group of the non-tumor derived cells (RAW 264.7, HUVEC) displayed more HSF-mediated signaling than the LC_50_ group. Altogether, these findings indicate that PDT induced extensive survival signaling in all cell types tested, whereby survival signaling was more prominent in cells treated by sublethal PDT.

#### NF-кB-mediated inflammatory response

NF-кB mediates an inflammatory response following PDT [35, 36]. As shown in Fig. 3, the transcription of various pro-inflammatory cytokines that are under the control of NF-кB, including interleukin 1A (*IL1A*), *IL1B*, *IL6*, and chemokine (C-X-C motif) ligand 8 (*CXCL8*), increased following PDT in the human cell types. Murine *Il1b* and *Cxcl2* were also considerably induced following PDT in RAW 264.7 cells. Sublethal PDT resulted in upregulation of vascular endothelial growth factor (*VEGF*) in SK-ChA-1 and A431 cells, which was downregulated in HUVEC cells. The pro-inflammatory factor prostaglandin-endoperoxide synthase 2 (*PTGS2*, *Ptgs2*) was also highly upregulated following PDT in HUVEC, RAW 264.7, and A431 cells.

#### Proteotoxic stress response

The proteotoxic stress response can be induced by ROS-mediated endoplasmic reticulum (ER) stress that leads to the accumulation of misfolded and unfolded proteins in the ER [37]. As a result, the UPR is initiated together with the activation of HSF1 [38]. ZPCL-PDT at both regimens induced upregulation of the UPR-associated genes DNA-damage-inducible transcript 3 (*DDIT3*, *Ddit3*), activating transcription factor 3 (*ATF3*, *Atf3*), protein phosphatase 1, and regulatory subunit 15A (*PPP1R15A*, *Ppp1r15a*) in all cell types (Fig. 3). PDT at LC_50_ triggered upregulation of DnaJ (Hsp40) homolog, subfamily B, member 9 (*DNAJB9*) in all cell types, of which the protein product protects cells from apoptosis [39]. With respect to HSF signaling, all cell types exhibited elevated *DNAJB1* (*Dnajb1*) and heat shock 70 kDa protein 1A (*HSPA1A*, *Hspa1a*) mRNA levels following PDT (Fig. 3). In contrast to RAW 264.7, SK-ChA-1, and A431 cells, HUVECs revealed a dose-dependent effect on the transcript levels of *HSPA1A*, *DNAJB1*, *JUN*, and *FOS*, where PDT at LC_90_ caused the most pronounced upregulation of these genes.

#### AP-1-mediated immediate early gene response

In response to various extracellular and intracellular (e.g., ROS) stimuli, the immediate early response is activated via apoptosis signal-regulating kinase 1 (ASK-1) that enables AP-1-mediated transcription [40]. The AP-1 transcription factors FBJ murine osteosarcoma viral oncogene homolog (*FOS*, *Fos*) and jun B proto-oncogene (*JUNB*, *Junb*) were upregulated in RAW 264.7, SK-ChA-1, and A431 cells in both the LC_50_ and LC_90_ groups (Fig. 3). Furthermore, the survival factor heparin-binding EGF-like growth factor (*HGEGF*, *Hbegf*) was strongly upregulated in all cell types following both PDT regimens. *EGFR* was downregulated in HUVEC, SK-ChA-1, and A431 cells, particularly in the LC_90_ group.

In addition, the effect of PDT on EGFR signaling in EGFR-overexpressing A431 cells versus SK-ChA-1 cells is shown in more detail in Fig. S4. This subanalysis revealed that PDT had an inhibitory effect on the various ErbB isoforms, which was observed in both cell lines, although *EGFR* (*ERBB1*) was mostly afflicted. Also, known downstream targets of EGFR [41] appeared to be more inhibited in A431 cells compared to SK-ChA-1 cells after supralethal PDT (Fig. S4).

#### HIF-1-mediated hypoxia-induced stress response

HIF-1 is a transcription factor that is induced by ROS and hypoxia [42], which promotes the transcription of genes involved in cell survival and angiogenesis [43]. ZPCL-PDT caused upregulation of various HIF-1-associated genes, including *VEGFA* (not in HUVECs), *PTGS2*, endothelin 1 (*EDN1*), myeloid cell leukemia 1 (*MCL1*), and phorbol-12-myristate-13-acetate-induced protein 1 (*PMAIP1*) (Fig. 3). The effects were more pronounced after sublethal PDT. PDT also upregulated several HIF-1-associated genes in RAW 264.7 cells, including *Vegfa*, *Ptgs2*, *Edn1*, and *Pmaip1* but not *Mcl1*. However, RAW 264.7 cells did not exhibit any dose-dependent differences as observed in the human cell types.

#### NFE2L2-mediated antioxidant response

The NFE2L2-mediated antioxidant response is activated by oxidative stress and serves to restore the cellular redox balance. As shown in Fig. 3, the NFE2L2 pathway was largely unaffected. In fact, PDT reduced the expression of genes involved in detoxification [e.g., ATP binding cassette subfamily C member 4 (*ABCC4*, *Abcc4*), ATP binding cassette subfamily G member 2 (*ABCG2*, *Abcg2*)] and antioxidant activity [e.g., epoxide hydrolase 1 (*EPHX1*, *Ephx1*)]. Heme oxygenase 1 (*HMOX1, Hmox1*) is linked to cell survival following PDT [44]. In addition to NFE2L2, HIF-1 (“HIF-1-mediated hypoxia-induced stress response”) is also able to mediate transcription of *HMOX1* [45]. Its gene expression after PDT was higher in RAW 264.7, SK-ChA-1, and A431 cells but not in HUVEC cells. This effect was more pronounced in the LC_50_ cells compared to the LC_90_ cells as evidenced by the log_2_ fold-changes in *HMOX1*/*Hmox1* gene expression: A431 (2.1 versus 1.3, respectively), SK-ChA-1 (2.1 versus 1.4, respectively), and RAW 264.7 cells (1.7 versus 1.5) respectively.

### PDT upregulates transcription-related proteins and downregulates proteins linked to EGFR signaling

To explore the cellular response in a cell line derived from a tumor that is refractory towards PDT [24], SK-ChA-1 cells were subjected to more in-depth analysis using an untargeted (phospho)proteomic-based approach 90 min after PDT. The EGFR-overexpressing A431 cell line was excluded from the (phospho)proteomic analysis to eliminate redundancy, given that SK-ChA-1 cells also express high basal levels of EGFR [46]. The differentially expressed phosphorylated and non-phosphorylated proteins (compared to non-treated cells) are presented in Table S5. A no-liposome, irradiation-only group was excluded because we have shown previously that red light irradiation has no effect on cells [14].

**Fig. 4:**
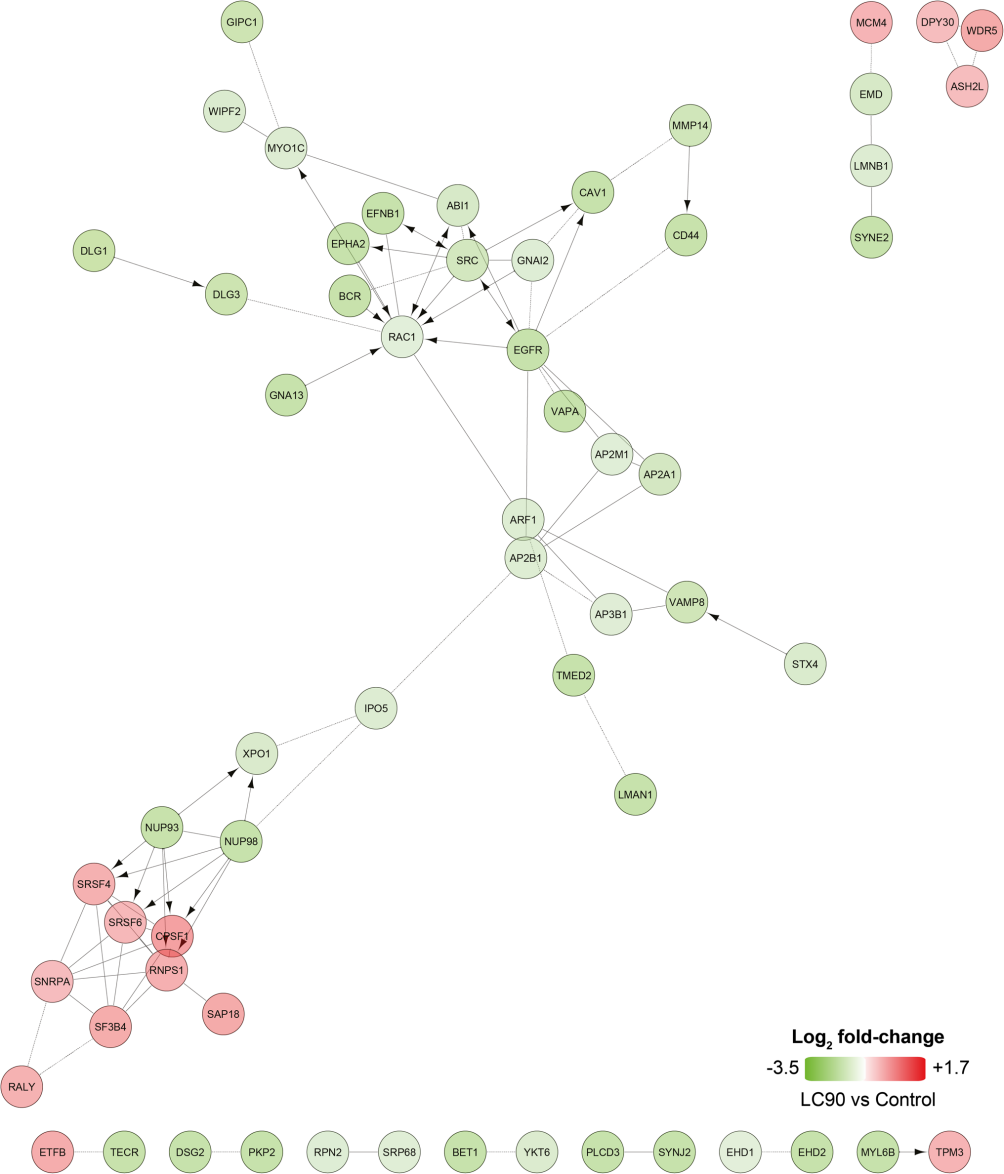
Differentially expressed proteins observed after ZPCL-PDT of SK-ChA-1 cells in the LC_90_ group. Upregulated (in *red*) and downregulated (in *green*) proteins between the PDT-treated groups and control group (*n* = 4 per group) were analyzed using Reactome to assess functional interactions [47, 48].* Arrows* indicate activating/catalyzing reactions, whereas* straight and dashed lines* indicate functional and predicted functional interactions, respectively. Proteins without functional interactions are not displayed in the figure

The proteome data revealed a dose-dependent response in the number of differentially expressed proteins (Fig. S5). To gain more insight in the affected molecular pathways, the data were analyzed with Reactome [47, 48] (Fig. 4). Based on the proteomics data, PDT caused downregulation of various proteins involved in endocytosis in the LC_50_ group, but more predominantly in the LC_90_ group [AP-2 complex subunit alpha-1 (AP2A1), AP2M1, AP2B1, AP3B1]. Furthermore, SK-ChA-1 cells that had been treated at LC_90_ upregulated proteins involved in pre-RNA splicing (serine/arginine-rich splicing factor 4 (SRSF4), SRSF6) and epigenetic control of transcription [protein dpy-30 homolog (DPY30), WD repeat-containing protein 5 (WDR5)] (Fig. 4).

**Fig. 5:**
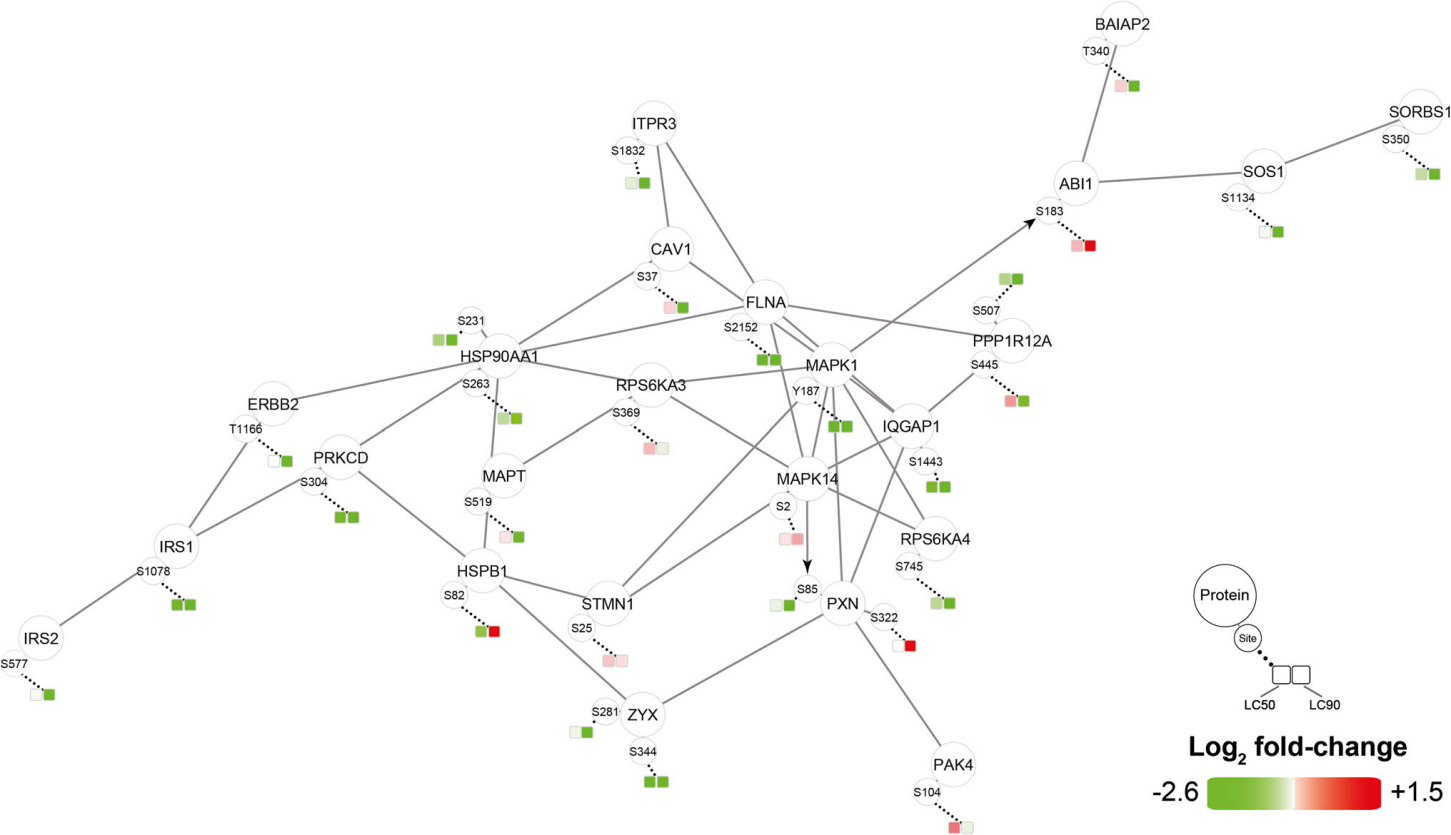
Phosphoproteomic analysis of SK-ChA-1 cells after ZPCL-PDT. The data (*n* = 4 per group) were analyzed with the Phosphopath plugin in Cytoscape [32]. Increased and decreased phosphorylation of proteins in the PDT-treated groups versus the control group are indicated in *red* and *green*, respectively. *Straight lines* and *arrows* indicate protein interactions (derived from the Biogrid database [78]) and kinase-substrate interactions (imported from PhosphoSitePlus [79]), respectively. Wikipathways was used for pathway analysis [80], where the dataset was queried against this database to identify pathways. For this figure, EGF, VEGF, insulin, FAK, and MAPK signaling pathways were selected

Phosphoproteomic data were analyzed with the Phosphopath plugin in Cytoscape [32] and only phosphosites which were differentially regulated in either LC_50_ or LC_90_ groups were analyzed. PDT of SK-ChA-1 cells induced phosphorylation of heat shock protein beta-1 (HSPB1) (Fig. 5), which is involved in the defense against oxidative stress [49, 50]. Furthermore, PDT decreased phosphorylation of proteins involved in EGFR signaling, such as mitogen-activated protein kinase 1 (MAPK1), son of sevenless homolog 1 (SOS1), and catenin delta-1 (CTNND1). This effect was more evident at LC_90_ inasmuch as these cells downregulated the EGFR-associated proteins EGFR (confirmed by Western blotting, Fig. S2), proto-oncogene tyrosine-protein kinase Src (SRC), caveolin-1 (CAV1), and phosphorylated proteins SOS1, related RAS viral (r-ras) oncogene homolog 2 (RRAS2), MAPK1, and MAPK3. Altogether, it seems that PDT induced the expression of transcription-related proteins and downregulated proteins involved in EGFR signaling.

### PDT affects metabolites that are involved in energy production and redox signaling

**Fig. 6:**
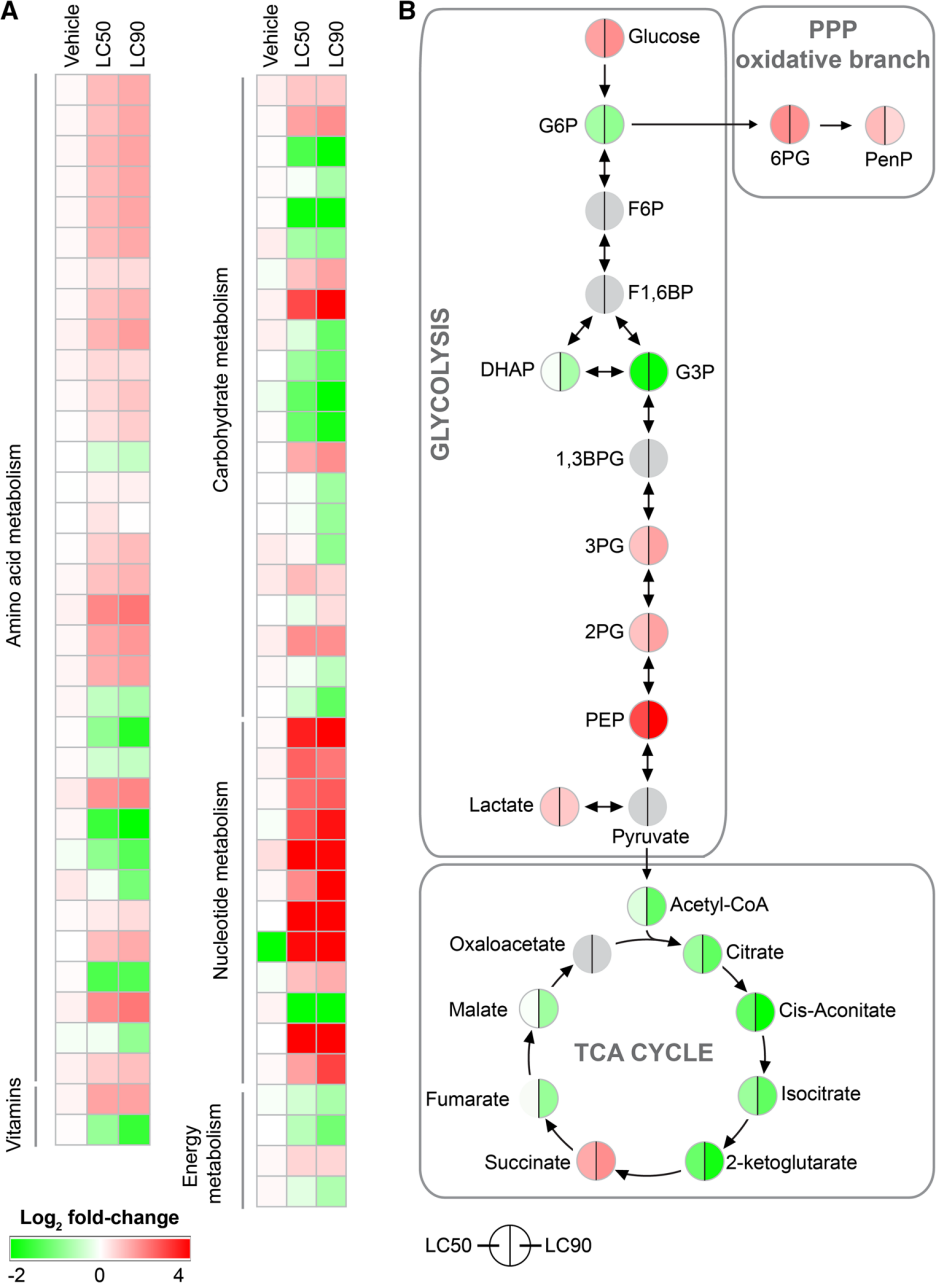
Metabolomic analysis of SK-ChA-1 cells after ZPCL-PDT. **a** Metabolites were classified into pathways and metabolite expression is depicted as the log_2_ fold-change (*bottom left corner*) between treated and control cells (*n* = 3 per group). Numerical values can be found in Table S6. **b** Log_2_ fold-change of metabolites in the category carbohydrate metabolism grouped per pathway. Changes in LC_50_- (*left*) and LC_90_-treated (*right*) SK-ChA-1 cells compared to control cells are depicted. Identical log_2_ fold-change values are plotted for 3PG and 2PG and for citrate and isocitrate, as these metabolites could not be resolved. Metabolites indicated in gray could not be quantified. TCA cycle, tricarboxylic acid cycle; PPP, pentose phosphate pathway; G6P, glucose-6-phosphate; F6P, fructose-6-phosphate; F1,6BP, fructose-1,6-bisphosphate; DHAP, dihydroxyacetone phosphate; G3P, glyceraldehyde-3-phosphate; 1,3 BPG, 1,3-bisphosphoglycerate; 3PG, 3-phosphoglycerate; 2PG, 2-phosphoglycerate; PEP, phosphoenolpyruvate; 6pG, 6-phosphogluconate; PenP, pentose-phosphate

Finally, PDT-treated SK-ChA-1 cells were investigated in terms of metabolomics at 90 min post-PDT. Incubation of cells with ZPCLs in the absence of light only marginally affected the metabolomic profile (Fig. 6a, Table S6), again confirming the in vitro safety of the ZPCLs.

PDT highly influenced almost all studied metabolites, whereby the effects observed in the LC_50_ group were essentially exacerbated in the LC_90_ group. PDT-subjected SK-ChA-1 cells upregulated multiple amino acid levels as well as metabolites involved in nucleotide metabolism. In contrast, metabolites involved in the TCA cycle and urea cycle were downregulated, reflecting perturbations in anaerobic energy production as evidenced by the lactate and succinate accumulation. Moreover, PDT also affected metabolites that modulate the redox balance (Fig. 6b). Glutathione and oxidized glutathione (GSSG) were downregulated, while nicotinamide adenine dinucleotide phosphate (NADP^+^) was upregulated. Possibly as a consequence of the pro-oxidative state, metabolites in the oxidative branch of the pentose phosphate pathway were upregulated (Table S6). Lastly, the nucleotide profile was also determined in PDT-treated cells, which showed slightly lower uridine triphosphate (UTP), cytidine triphosphate (CTP), and guanosine triphosphate (GTP) levels in the LC_50_ group (Fig. S6). The lower ATP:ADP ratio in PDT-treated cells may be indicative of dying cells (Fig. S6).

## Discussion

Clinical PDT may be enhanced by pharmacologically interfering in molecular pathways that mediate resistance to therapy [2]. During PDT, light intensity attenuates in the tumor tissue as a result of absorption and scattering, creating a gradient of cumulative light dose (fluence) across the tumor. Since PDT-mediated ROS production is proportional to the fluence [14], tumor cells that are more distal from the light source, or tumor cells that are insufficiently oxygenated, may experience less oxidative stress than fully exposed and oxygenated cells [23], allowing the sublethally afflicted cells to activate survival pathways. Inasmuch as tumor cell survival may ultimately enable recurrence and metastasis, it is critical that the tumor bulk is completely eradicated in a single PDT session.

One potential strategy to optimize PDT is using pharmacological adjuvants that inhibit post-PDT survival signaling, which may be co-administered with the photosensitizer [11–13]. This study was conducted to determine which pathways are activated and hence eligible for pharmacological targeting. The response of tumor parenchymal and non-parenchymal cells subjected to sublethal (LC_50_) and supralethal PDT (LC_90_) was therefore investigated in the acute phase of PDT—a time point where the transcriptome and acute phase proteins were expected to be dysregulated. SK-ChA-1 and A431 cells were used because the former are derived from a tumor known to be refractory to PDT [51, 52] and because both overexpress EGFR, which was shown to be profoundly affected by PDT. It is critical to underscore that the post-PDT environment temporally evolves in a dynamic manner at the level of the transcriptome, lipidome, proteome, and metabolome [53]. In support of this, the extent of PDT-induced cell death progressively increased at 2, 4, and 24 h after PDT and transcriptomic and (phospho)proteomic analysis revealed that mRNA and protein expression was discordant at 90 min post-PDT (Fig. S7). First, mRNA and protein expression profiles may be more in sync at later time points, *i.e.*, when the mRNA has been translated to functional proteins. Second, the transcriptome and proteome are also expected to change over time, potentially necessitating an acclimating pharmacological inhibition strategy after PDT. Because the transcriptomic-, (phospho)proteomic-, and metabolomic temporal changes are vital to therapeutic outcome, studies in our labs are underway to establish post-PDT molecular signatures across the 24-hour time span.

In the acute phase, transcriptomic analysis revealed that PDT-treated tumor cells (SK-ChA-1, A431) were afflicted at multiple physiological and biochemical junctions and activated extensive survival signaling via HIF-1, NF-кB, AP-1, and HSF. Survival signaling was most pronounced in the low-dose PDT group, which is detrimental to the desired clinical outcome. Second, PDT-treated SK-ChA-1 cells downregulated proteins involved in EGFR signaling. Third, metabolomic analysis of PDT-treated SK-ChA-1 cells pointed to downregulation of metabolites involved in energy metabolism (glycolysis, TCA cycle), altered cellular redox state, and upregulation of metabolites involved in nucleotide metabolism and the pentose phosphate pathway. These latter two findings are expected to be beneficial for PDT outcome, as EGFR downregulation and perturbed energy metabolism negatively affect cell viability and proliferation and hence offset the survival signaling.

The ROAST gene set analysis supports our hypothesis that suboptimally treated tumor cells (LC_50_) engage in more extensive survival signaling in response to PDT. Especially the HIF-1- and NF-кB-mediated pathways may be attractive for therapeutic interventions. PDT of SK-ChA-1 and A431 cells upregulated genes downstream of HIF-1 and NF-кB (*IL1A*, *IL1B*, *IL6*, *CXCL8*, *VEGFA*, *HMOX1*) that mediate inflammation, survival, and angiogenesis [54, 55]. These findings have been echoed in literature (Table 1). Whereas overexpression of HIF-1 was associated with therapeutic resistance in 5-aminolevulinic acid (5-ALA)-PDT-treated human esophageal carcinoma cells [56], combination therapy of siRNA-mediated knockdown of HIF-1 with Photosan-PDT significantly improved therapeutic efficacy in human head-and-neck cancer (SCC4, SAS) tumor-bearing mice [57]. Corroboratively, treatment of A431 and SK-ChA-1 cells with the HIF-1 inhibitor acriflavine significantly improved PDT efficacy [11, 13]. Similarly, it was shown in various studies that combined treatment comprising NF-кB inhibitors and PDT augmented therapeutic efficacy [35, 58, 59].

**Table 1:** Potential druggable targets that were identified in this study

	Identified target	Druggable target	General function	Inhibitor	PDT efficacy	References
Transcriptomics	↑ *HMOX1*	HMOX1	Cytoprotective, antioxidative properties	SnPPIX	↑	[75]
				ZnPPIX	↑	[44, 76]
	↑ AP-1 pathway	AP-1	Proliferation, inflammation, apoptosis	–	n.d.	[9]
	↑ HIF-1 pathway	HIF-1	Survival, angiogenesis, glycolysis	Acriflavine	↑	[11, 13]
				HIF-1α siRNA	↑	[57]
	↑ HSF pathway	HSF1	Proteostasis, survival	–	n.d.	[9]
	↑ NF-κB pathway	NF-κB	Inflammation, proliferation, anti-apoptosis	NF-κB siRNA	↑	[35]
				Dihydroartemisinin	↑	[58]
				BAY 11-7082	↑	[59]
Proteomics	↑ HSPB1	HSPB1	Anti-apoptosis, cell invasion	–	↑ ↓	[50, 77]
Metabolomics	↑ Succinate	SUCNR1	Inflammation, HIF-1 stabilization	–	n.d.	[73]

For all the molecular targets, its general function is listed, as well as whether inhibition improves (indicated with ↑) or hampers (indicated with ↓) PDT efficacy

*SnPPIX* tin protoporphyrin, *ZnPPIX* zinc protoporphyrin, *siRNA* small interfering RNA, *n.d.* not determined, *SUCNR1* succinate receptor 1

In addition to the tumor-derived cell lines, murine macrophages (RAW 264.7) responded fervently to PDT, inasmuch as these cells significantly upregulated all survival pathways (except for NFE2L2 in the LC_90_ group). This hyperactive state may in part have been caused by the fact that macrophages become activated upon exposure to dying cells and cell debris [60], including post-PDT [35]. The same pattern was observed for HUVEC cells, but in contrast to the tumor cell lines, only few differences were observed between the LC_50_ and LC_90_ groups. Unexpectedly, after PDT the endothelial cells slightly downregulated *VEGF*, which is a growth factor for (tumor) endothelium that stimulates angiogenesis. Zhang et al. also observed downregulated VEGF protein levels after hypericin-PDT in HUVECs [61], which may indicate that PDT is able to induce growth inhibition of tumor endothelium.

**Fig. 7:**
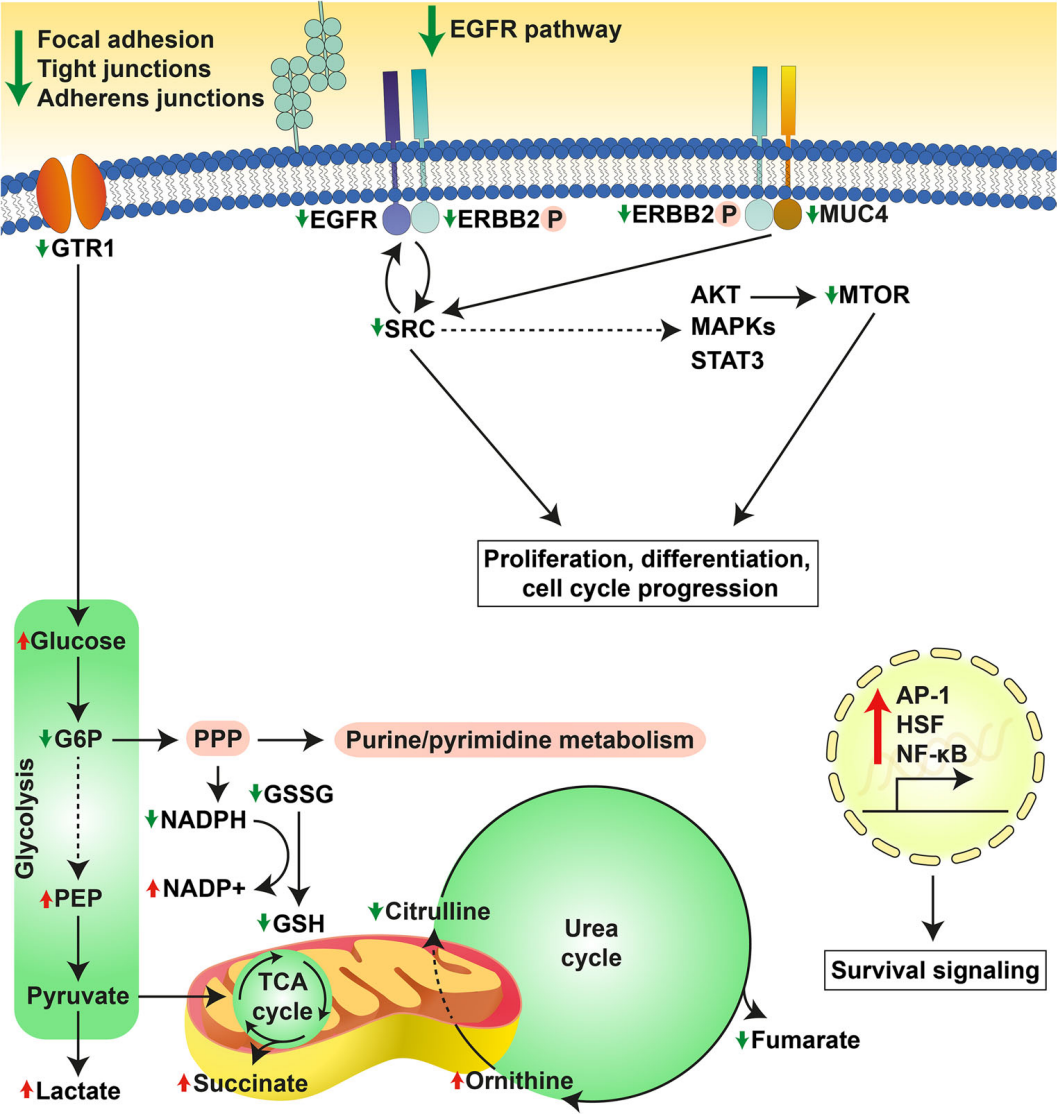
Overview of the cellular response of SK-ChA-1 cells to supralethal (LC_90_) PDT. In response to PDT, SK-ChA-1 cells downregulate proteins involved in focal adhesion, tight and adherens junctions, and EGFR signaling. Metabolic processes that are dependent on mitochondria (TCA cycle, urea cycle) appear to be downmodulated, whereas the antioxidant response was activated. On the transcriptomic level, SK-ChA-1 cells exhibited upregulation of AP-1-, HSF-, and NF-кB-mediated signaling that may contribute to cell survival. *Straight* and *dashed arrows* indicate direct and indirect reactions, respectively. Phosphorylated proteins are indicated with (P)

SK-ChA-1 cells were also subjected to (phospho)proteomic and metabolomic analysis, of which the main results are summarized in Fig. 7. At the proteomic level, PDT-mediated phosphorylation of HSPB1, which is a stress protein that acts as a chaperone to stimulate survival under stress conditions [50]. PDT at LC_90_ downregulated proteins involved in focal adhesion [CAV1, integrin alpha-2 (ITA2)], adherens junctions (CTNND1, EpCAM), and tight junctions (phosphorylated ZO1 and ZO3). As reported in [62–64], PDT may oxidatively damage proteins involved in cell–cell adhesion, cytoskeletal structure, and focal adhesion, which appears to be dependent on cell type, photosensitizer concentration, and light dose. However, it may also contribute to a higher metastatic potential after PDT, inasmuch as loss of adhesion proteins is associated with invasion [62]. Further research is warranted to establish whether PDT enhances the metastatic potential of cancer cells, as activation of both survival and metastasis pathways by PDT may hamper clinical safety of the procedure.

Supralethal PDT also downregulated various proteins involved in EGFR signaling, which is an important therapeutic target as it is overexpressed in numerous cancer types [19]. Previous studies have shown that SK-ChA-1 and A431 cells in the absence of PDT are sensitive to EGFR inhibitors, as these compounds inhibited cell growth [46, 65]. ZPCL-PDT of SK-ChA-1 cells at LC_90_ revealed downregulation of EGFR on both the transcriptomic and proteomic level. Both SK-ChA-1 and A431 cells exhibited a reduction in *EGFR* mRNA levels after PDT and this effect was enhanced in the LC_90_ group. Although the exact mechanism is still unknown, the general trend is that PDT is able to inhibit and/or degrade EGFR, thereby deterring tumor growth and inducing apoptosis [66]. However, exceptions do exist. For instance, Edmonds and co-workers showed that human ovarian carcinoma (OVCAR-5) and non-small cell lung cancer (H460) cell lines upregulated EGFR after PDT with verteporfin (log *P* = 3.74) [67, 68]. Inhibition of EGFR with erlotinib increased PDT efficacy and resulted in apoptotic cell death [67], linking pharmacological EGFR inhibition to cell demise. Also, a more recent study demonstrated that erlotinib treatment prior to PDT induced higher complete response rates in NSCLC (H460, A549)-xenografted mice [69]. Interestingly, treatment of various cancer cell lines with the photosensitizer Photofrin (porfimer sodium, log *P* = 8.5 [2]) alone downregulated EGFR protein expression, which was enhanced upon PDT, indicating that Photofrin alone is able to downmodulate EGFR expression [70]. ZnPC is a highly lipophilic photosensitizer (log *P* = 8.5 [2]) that intercalates into biomembranes [13]. Given that EGFR is a transmembrane protein, ZnPC is expected to reside in the direct vicinity of the transmembrane domain of EGFR, where it can subsequently cause oxidative modification of EGFR’s transmembrane structures and impede its functional properties. The same applies to verteporfin and Photofrin. However, apparently the site of ROS generation is not ubiquitously linked to protein dysfunctionalization. Instead, EGFR expression after PDT is photosensitizer-dependent, whereby inhibition of EGFR by PDT may contribute to an anti-cancer effect when photosensitizers are employed that induce its downregulation, such as ZnPC, by an as yet undefined mechanism.

The metabolomics data of PDT-treated SK-ChA-1 cells showed similar trends between the LC_50_ and LC_90_ groups, although the effects were more pronounced in the LC_90_ group. PDT-treated cells exhibited increased glucose whereas a number of glycolysis-associated metabolites were reduced, suggesting that glucose is shuttled into pathways that branch off glycolysis, such as the pentose phosphate pathway. Also, the TCA cycle appeared to be downregulated following PDT, as evidenced by downregulation of acetyl-CoA, citrate, α-ketoglutarate, and malate. As a result of ROS production during PDT, the redox status of a cell may be seriously affected. This is also observed in PDT-treated SK-ChA-1 cells, as regulators of the redox response differed (e.g., reduction of glutathione and GSSG, increase in NADP^+^). The post-PDT pro-oxidative state may also explain the upregulation of the pentose phosphate pathway (Fig. 6b), as the pentose phosphate pathway contributes to the production of NADPH—a major player in the antioxidant response [71]. Another important factor that was increased in PDT-treated SK-ChA-1 cells is succinate. Mitochondria are a known target of ZnPC-based PDT [2, 17], after which mitochondria-localized succinate may be released into the cytoplasm [72]. Succinate has been shown to mediate ATP generation in mitochondria, activation of HIF-1, and pro-inflammatory signaling (reviewed in [73]). Pharmacological strategies that limit succinate production could therefore serve as a strategy to augment PDT efficacy, although succinate build-up in mitochondria is also a precursor condition for latent oxidative stress [74] that in turn may promote tumor cell death.

To our knowledge, this is the first study that explored the PDT response in such detail at the cellular and molecular level. Therefore, it may provide novel information that could be valuable to design new therapeutic strategies, possibly based on therapeutic targets that were found in this study (Table 1). Consistent with earlier reports, the combined use of PDT and inhibitors of survival pathways may be an attractive approach to improve therapeutic efficacy in the aforementioned clinically recalcitrant cancer types.

### Electronic supplementary material

Below is the link to the electronic supplementary material.

Supplementary material 1 (PDF 5335 kb)

Supplementary material 2 (XLSX 12 kb)

Supplementary material 3 (XLSX 24 kb)

Supplementary material 4 (XLSX 11 kb)

Supplementary material 5 (XLSX 19 kb)

Supplementary material 6 (XLSX 162 kb)

Supplementary material 7 (XLSX 17 kb)
