# Supplementary material for: Multi-OMIC profiling of survival and metabolic signaling networks in cells subjected to photodynamic therapy
Source: Cell Mol Life Sci. 2016 Nov 1;74(6):1133–51. doi: 10.1007/s00018-016-2401-0 (PMC5309296; doi:10.1007/s00018-016-2401-0)
Supplement: Supplementary file 1 — Supplementary material 1 (PDF 5335 kb) [file 18_2016_2401_MOESM1_ESM.pdf]

Supplementary material

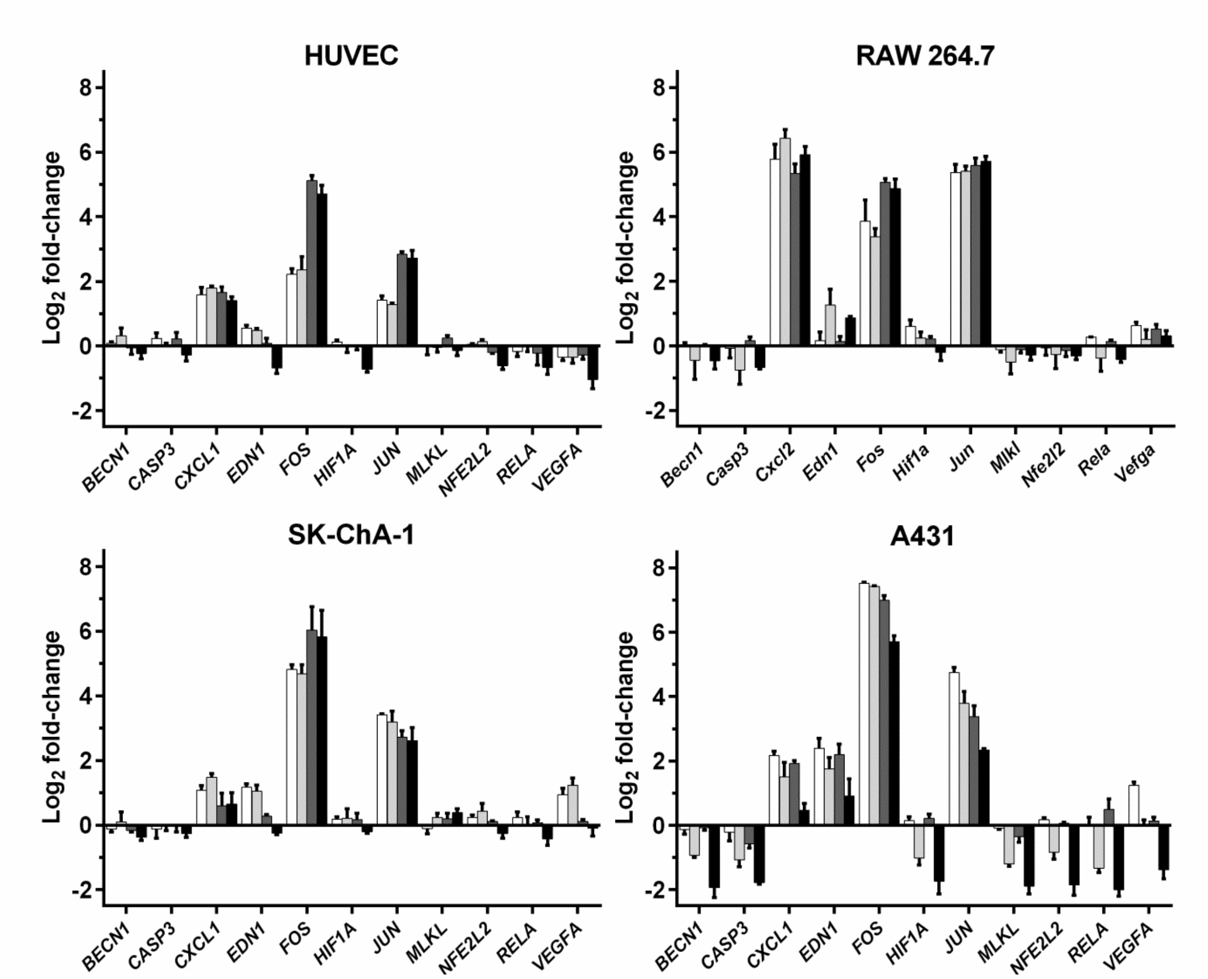

Supplementary Figure S1. Microarray validation by qRT-PCR. The expression levels of a panel of genes for both the LC_{50} (white bars) and LC_{90} group (dark grey bars bars) as assessed by microarray were validated by qRT-PCR. The corresponding qRT-PCR data are depicted for the LC_{50} group in light grey and the LC_{90} group in black. Gene expression is depicted as the log_2 fold-change between PDT-treated and control cells. qRT-PCR data were normalized to the expression level of the reference gene *RPS18*.

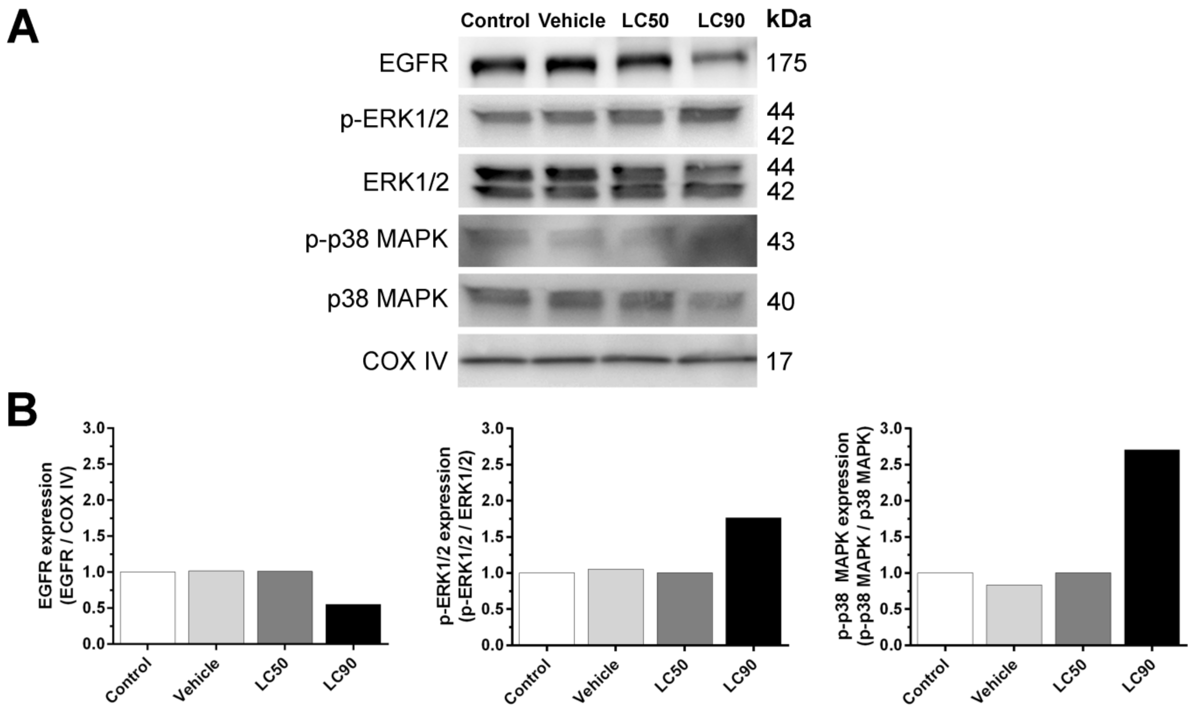

Supplementary Figure S2. Western Blot analysis of SK-ChA-1 cells after ZPCL-PDT. (A) Western Blot analysis of a panel of proteins that were differentially expressed after (phospho)proteomics. Western Blot analysis revealed similar trends for EGFR and p-p38 MAPK as obtained by (phospho)proteomics. While p-ERK1 (MAPK3) was detected by Western Blot, SK-ChA-1 cells did not exhibit any detectable p-ERK2 (MAPK1) levels. (B) Protein bands were quantified using ImageJ software and normalized to total protein (COX IV for EGFR, and total ERK and total p38 MAPK for their corresponding phosphorylated forms).

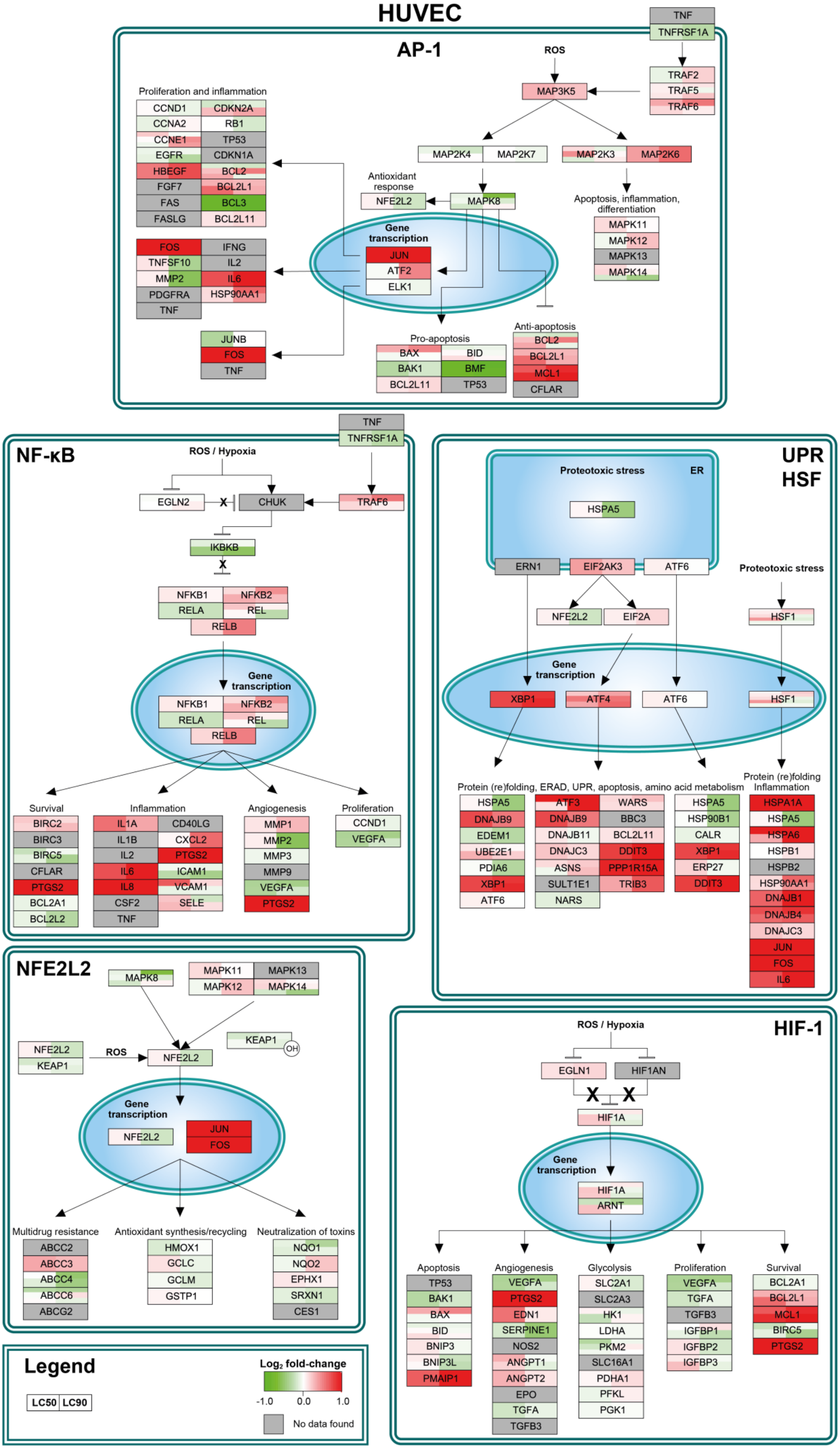
HUVEC
AP-1
TNF
TNFRSF1A
ROS
MAP3K5
TRAF2
TRAF5
TRAF6
Proliferation and inflammation
CCND1
CDKN2A
CCNA2
RB1
CCNE1
TP53
EGFR
CDKN1A
HBEGF
BCL2
FGF7
BCL2L1
FAS
BCL3
FASLG
BCL2L11
MAP2K4
MAP2K7
MAP2K3
MAP2K6
Antioxidant response
NFE2L2
MAPK8
Apoptosis, inflammation, differentiation
MAPK11
MAPK12
MAPK13
MAPK14
Gene transcription
JUN
ATF2
ELK1
FOS
IFNG
TNFSF10
IL2
MMP2
IL6
PDGFRA
HSP90AA1
TNF
JUNB
FOS
TNF
Pro-apoptosis
BAX
BID
BAK1
BMF
BCL2L11
TP53
Anti-apoptosis
BCL2
BCL2L1
MCL1
CFLAR
NF-κB
ROS / Hypoxia
TNF
TNFRSF1A
EGLN2
CHUK
TRAF6
IKBKB
NFKB1
NFKB2
RELA
REL
RELB
Gene transcription
NFKB1
NFKB2
RELA
REL
RELB
Survival
BIRC2
BIRC3
BIRC5
CFLAR
PTGS2
BCL2A1
BCL2L2
Inflammation
IL1A
CD40LG
IL1B
CXCL2
IL2
PTGS2
IL6
ICAM1
IL8
VCAM1
CSF2
SELE
TNF
Angiogenesis
MMP1
MMP2
MMP3
MMP9
VEGFA
PTGS2
Proliferation
CCND1
VEGFA
UPR
HSF
Proteotoxic stress
ER
HSPA5
ERN1
EIF2AK3
ATF6
Proteotoxic stress
NFE2L2
EIF2A
HSF1
Gene transcription
XBP1
ATF4
ATF6
HSF1
Protein (re)folding, ERAD, UPR, apoptosis, amino acid metabolism
Protein (re)folding
Inflammation
HSPA5
DNAJB9
EDEM1
UBE2E1
PDIA6
XBP1
ATF6
ATF3
WARS
DNAJB9
BBC3
DNAJB11
BCL2L11
DNAJC3
DDIT3
ASNS
PPP1R15A
SULT1E1
TRIB3
NARS
HSPA5
HSP90B1
CALR
XBP1
ERP27
DDIT3
HSPA1A
HSPA5
HSPA6
HSPB1
HSPB2
HSP90AA1
DNAJB1
DNAJB4
DNAJC3
JUN
FOS
IL6
NFE2L2
MAPK8
MAPK11
MAPK13
MAPK12
MAPK14
KEAP1
OH
NFE2L2
KEAP1
ROS
NFE2L2
Gene transcription
NFE2L2
JUN
FOS
Multidrug resistance
ABCC2
ABCC3
ABCC4
ABCC6
ABCG2
Antioxidant synthesis/recycling
HMOX1
GCLC
GCLM
GSTP1
Neutralization of toxins
NQO1
NQO2
EPHX1
SRXN1
CES1
HIF-1
ROS / Hypoxia
EGLN1
HIF1AN
HIF1A
Gene transcription
HIF1A
ARNT
Apoptosis
TP53
BAK1
BAX
BID
BNIP3
BNIP3L
PMAIP1
Angiogenesis
VEGFA
PTGS2
EDN1
SERPINE1
NOS2
ANGPT1
ANGPT2
EPO
TGFA
TGFB3
Glycolysis
SLC2A1
SLC2A3
HK1
LDHA
PKM2
SLC16A1
PDHA1
PFKL
PGK1
Proliferation
VEGFA
TGFA
TGFB3
IGFBP1
IGFBP2
IGFBP3
Survival
BCL2A1
BCL2L1
MCL1
BIRC5
PTGS2
Legend
LC50
LC90
Log₂ fold-change
-1.0
0.0
1.0
No data found

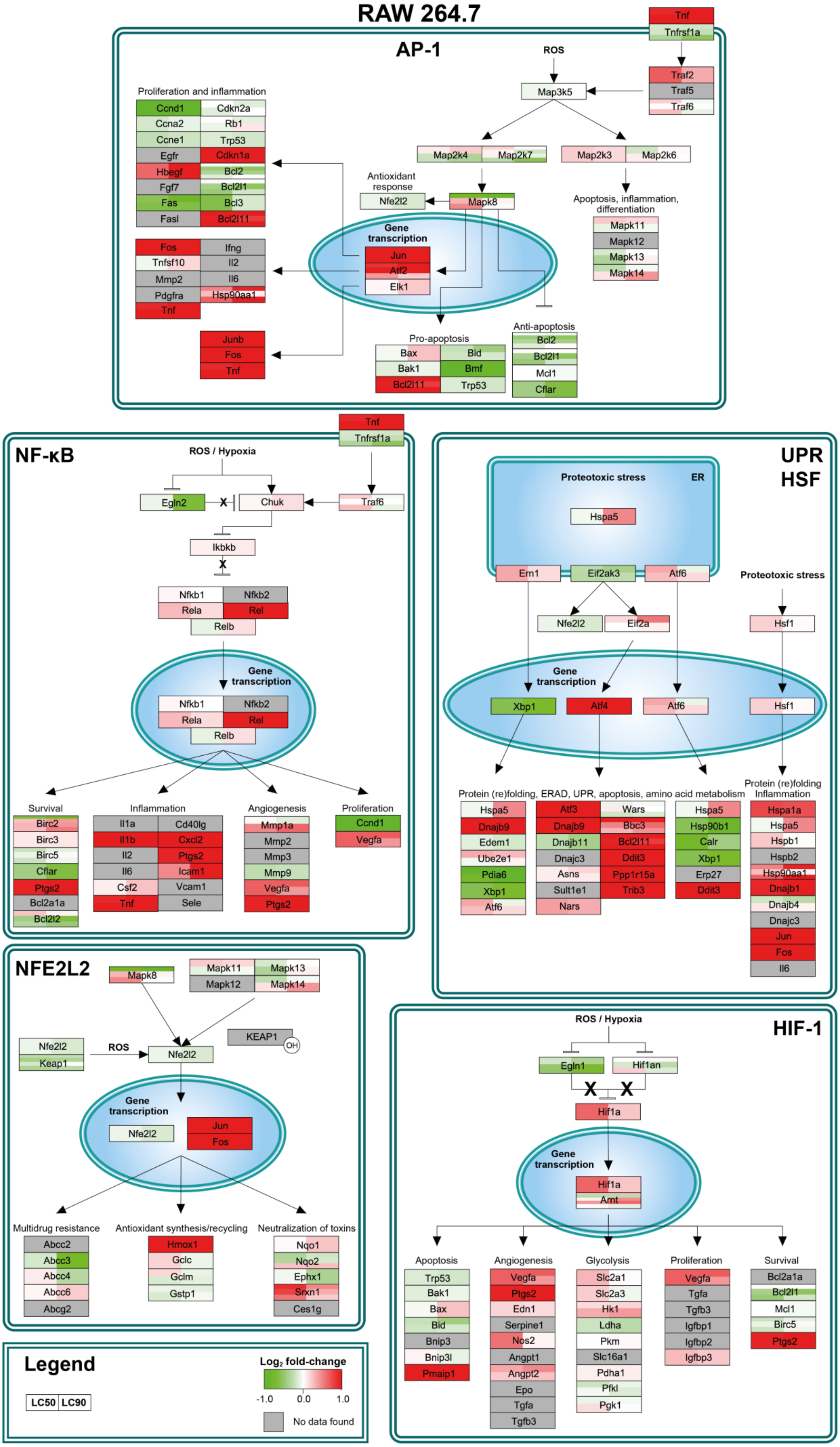
RAW 264.7
AP-1
Tnf
Tnfrsf1a
ROS
Traf2
Traf5
Traf6
Map3k5
Proliferation and inflammation
Ccnd1
Cdkn2a
Ccna2
Rb1
Ccne1
Trp53
Egfr
Cdkn1a
Hbegf
Bcl2
Fgf7
Bcl2l1
Fas
Bcl3
Fasl
Bcl2l11
Map2k4
Map2k7
Map2k3
Map2k6
Antioxidant response
Nfe2l2
Mapk8
Apoptosis, inflammation, differentiation
Mapk11
Mapk12
Mapk13
Mapk14
Gene transcription
Jun
Atf2
Elk1
Fos
Ifng
Tnfsf10
Il2
Mmp2
Il6
Pdgfra
Hsp90aa1
Tnf
Junb
Fos
Tnf
Pro-apoptosis
Bax
Bid
Bak1
Bmf
Bcl2l11
Trp53
Anti-apoptosis
Bcl2
Bcl2l1
Mcl1
Cflar
NF-κB
Tnf
Tnfrsf1a
ROS / Hypoxia
Egln2
Chuk
Traf6
Ikbkb
Nfkb1
Nfkb2
Rela
Rel
Relb
Gene transcription
Nfkb1
Nfkb2
Rela
Rel
Relb
Survival
Birc2
Birc3
Birc5
Cflar
Ptgs2
Bcl2a1a
Bcl2l2
Inflammation
Il1a
Cd40lg
Il1b
Cxcl2
Il2
Ptgs2
Il6
Icam1
Csf2
Vcam1
Tnf
Sele
Angiogenesis
Mmp1a
Mmp2
Mmp3
Mmp9
Vegfa
Ptgs2
Proliferation
Ccnd1
Vegfa
UPR
HSF
Proteotoxic stress
ER
Hspa5
Ern1
Eif2ak3
Atf6
Proteotoxic stress
Nfe2l2
Eif2a
Hsf1
Gene transcription
Xbp1
Atf4
Atf6
Hsf1
Protein (re)folding, ERAD, UPR, apoptosis, amino acid metabolism
Hspa5
Dnajb9
Edem1
Ube2e1
Pdia6
Xbp1
Atf6
Atf3
Wars
Dnajb9
Bbc3
Dnajb11
Bcl2l11
Dnajc3
Ddit3
Asns
Ppp1r15a
Sult1e1
Trib3
Nars
Hspa5
Hsp90b1
Calr
Xbp1
Erp27
Ddit3
Protein (re)folding
Inflammation
Hspa1a
Hspa5
Hspb1
Hspb2
Hsp90aa1
Dnajb1
Dnajb4
Dnajc3
Jun
Fos
Il6
NFE2L2
Mapk8
Mapk11
Mapk13
Mapk12
Mapk14
KEAP1
OH
Nfe2l2
Keap1
ROS
Nfe2l2
Gene transcription
Nfe2l2
Jun
Fos
Multidrug resistance
Abcc2
Abcc3
Abcc4
Abcc6
Abcg2
Antioxidant synthesis/recycling
Hmox1
Gclc
Gclm
Gstp1
Neutralization of toxins
Nqo1
Nqo2
Ephx1
Srxn1
Ces1g
HIF-1
ROS / Hypoxia
Egln1
Hif1an
X
X
Hif1a
Gene transcription
Hif1a
Arnt
Apoptosis
Trp53
Bak1
Bax
Bid
Bnip3
Bnip3l
Pmaip1
Angiogenesis
Vegfa
Ptgs2
Edn1
Serpine1
Nos2
Angpt1
Angpt2
Epo
Tgfa
Tgfb3
Glycolysis
Slc2a1
Slc2a3
Hk1
Ldha
Pkm
Slc16a1
Pdha1
Pfkl
Pgk1
Proliferation
Vegfa
Tgfa
Tgfb3
Igfbp1
Igfbp2
Igfbp3
Survival
Bcl2a1a
Bcl2l1
Mcl1
Birc5
Ptgs2
Legend
LC50
LC90
Log₂ fold-change
-1.0
0.0
1.0
No data found

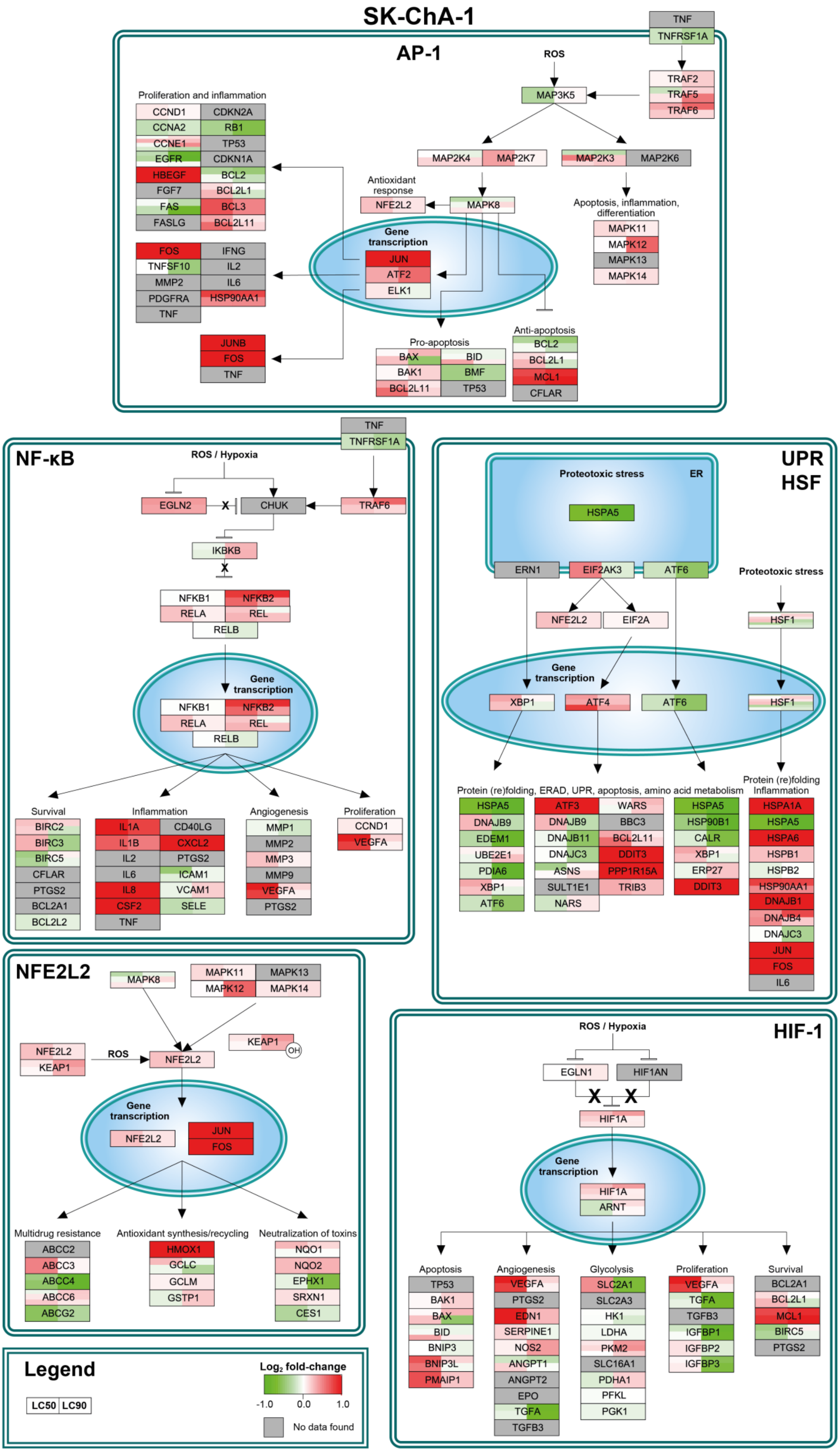
SK-ChA-1
AP-1
TNF
TNFRSF1A
ROS
TRAF2
TRAF5
TRAF6
MAP3K5
Proliferation and inflammation
CCND1 CDKN2A
CCNA2 RB1
CCNE1 TP53
EGFR CDKN1A
HBEGF BCL2
FGF7 BCL2L1
FAS BCL3
FASLG BCL2L11
MAP2K4 MAP2K7
MAP2K3 MAP2K6
Antioxidant response
NFE2L2
MAPK8
Apoptosis, inflammation, differentiation
MAPK11
MAPK12
MAPK13
MAPK14
Gene transcription
JUN
ATF2
ELK1
FOS IFNG
TNFSF10 IL2
MMP2 IL6
PDGFRA HSP90AA1
TNF
JUNB
FOS
TNF
Pro-apoptosis
BAX BID
BAK1 BMF
BCL2L11 TP53
Anti-apoptosis
BCL2
BCL2L1
MCL1
CFLAR
NF-κB
ROS / Hypoxia
TNF
TNFRSF1A
EGLN2
CHUK
TRAF6
IKBKB
NFKB1 NFKB2
RELA REL
RELB
Gene transcription
NFKB1 NFKB2
RELA REL
RELB
Survival
BIRC2
BIRC3
BIRC5
CFLAR
PTGS2
BCL2A1
BCL2L2
Inflammation
IL1A CD40LG
IL1B CXCL2
IL2 PTGS2
IL6 ICAM1
IL8 VCAM1
CSF2 SELE
TNF
Angiogenesis
MMP1
MMP2
MMP3
MMP9
VEGFA
PTGS2
Proliferation
CCND1
VEGFA
UPR
HSF
Proteotoxic stress
ER
HSPA5
ERN1 EIF2AK3 ATF6
Proteotoxic stress
NFE2L2 EIF2A
HSF1
Gene transcription
XBP1 ATF4 ATF6 HSF1
Protein (re)folding, ERAD, UPR, apoptosis, amino acid metabolism
HSPA5
DNAJB9
EDEM1
UBE2E1
PDIA6
XBP1
ATF6
ATF3 WARS
DNAJB9 BBC3
DNAJB11 BCL2L11
DNAJC3 DDIT3
ASNS PPP1R15A
SULT1E1 TRIB3
NARS
HSPA5
HSP90B1
CALR
XBP1
ERP27
DDIT3
Protein (re)folding
Inflammation
HSPA1A
HSPA5
HSPA6
HSPB1
HSPB2
HSP90AA1
DNAJB1
DNAJB4
DNAJC3
JUN
FOS
IL6
NFE2L2
MAPK8
MAPK11 MAPK13
MAPK12 MAPK14
KEAP1
OH
NFE2L2
KEAP1
ROS
NFE2L2
Gene transcription
NFE2L2
JUN
FOS
Multidrug resistance
ABCC2
ABCC3
ABCC4
ABCC6
ABCG2
Antioxidant synthesis/recycling
HMOX1
GCLC
GCLM
GSTP1
Neutralization of toxins
NQO1
NQO2
EPHX1
SRXN1
CES1
HIF-1
ROS / Hypoxia
EGLN1 HIF1AN
HIF1A
Gene transcription
HIF1A
ARNT
Apoptosis
TP53
BAK1
BAX
BID
BNIP3
BNIP3L
PMAIP1
Angiogenesis
VEGFA
PTGS2
EDN1
SERPINE1
NOS2
ANGPT1
ANGPT2
EPO
TGFA
TGFB3
Glycolysis
SLC2A1
SLC2A3
HK1
LDHA
PKM2
SLC16A1
PDHA1
PFKL
PGK1
Proliferation
VEGFA
TGFA
TGFB3
IGFBP1
IGFBP2
IGFBP3
Survival
BCL2A1
BCL2L1
MCL1
BIRC5
PTGS2
Legend
LC50 LC90
Log₂ fold-change
-1.0 0.0 1.0
No data found

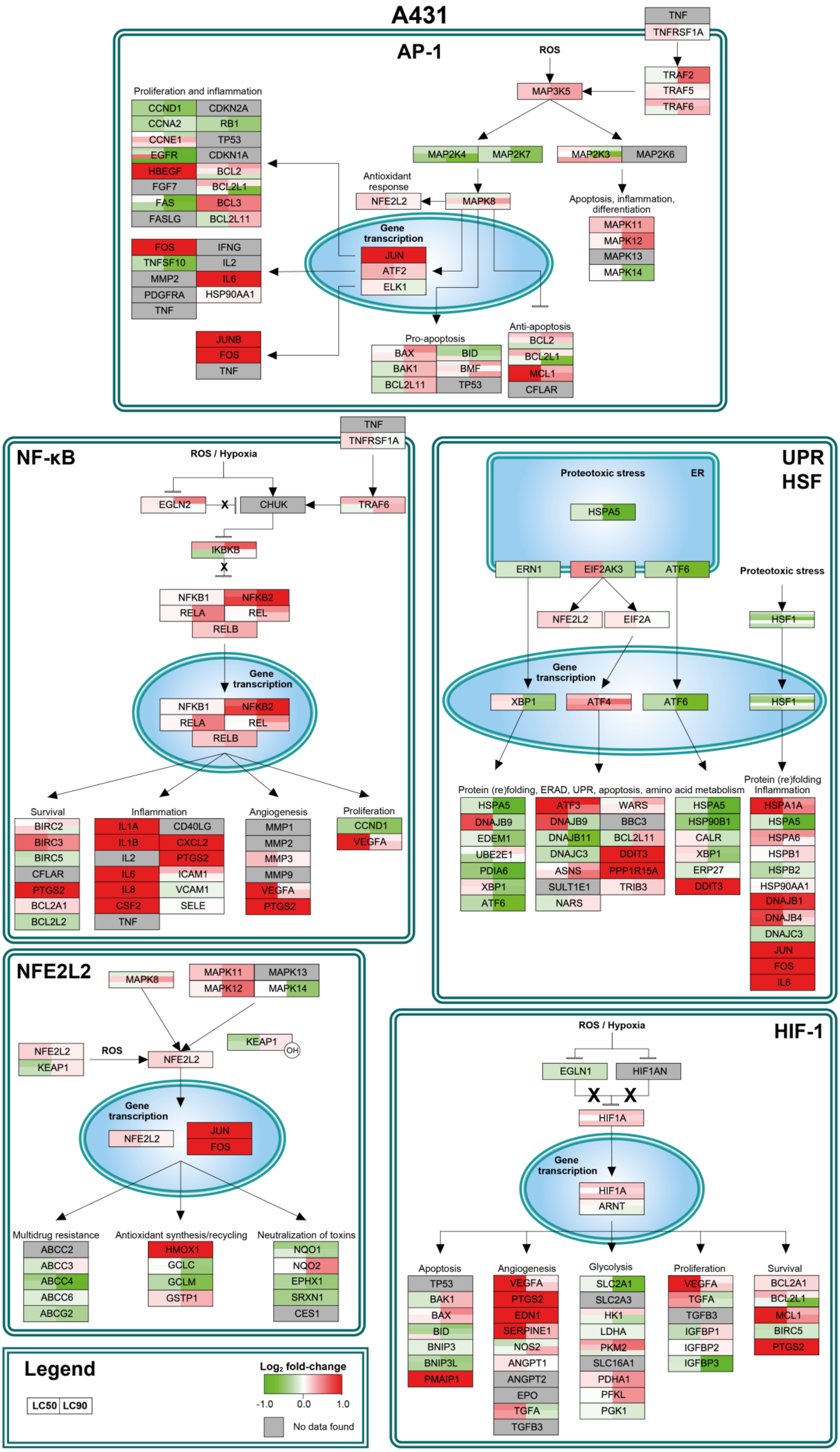
A431
TNF
TNFRSF1A
AP-1
ROS
TRAF2
TRAF5
TRAF6
MAP3K5
MAP2K4
MAP2K7
MAP2K3
MAP2K6
Proliferation and inflammation
CCND1
CDKN2A
CCNA2
RB1
CCNE1
TP53
EGFR
CDKN1A
HBEGF
BCL2
FGF7
BCL2L1
FAS
BCL3
FASLG
BCL2L11
FOS
IFNG
TNFSF10
IL2
MMP2
IL6
PDGFRA
HSP90AA1
TNF
JUNB
FOS
TNF
Antioxidant response
NFE2L2
MAPK8
Gene transcription
JUN
ATF2
ELK1
Apoptosis, inflammation, differentiation
MAPK11
MAPK12
MAPK13
MAPK14
Pro-apoptosis
BAX
BID
BAK1
BMF
BCL2L11
TP53
Anti-apoptosis
BCL2
BCL2L1
MCL1
CFLAR
NF-κB
ROS / Hypoxia
TNF
TNFRSF1A
EGLN2
CHUK
TRAF6
IKBKB
NFKB1
NFKB2
RELA
REL
RELB
Gene transcription
NFKB1
NFKB2
RELA
REL
RELB
Survival
BIRC2
BIRC3
BIRC5
CFLAR
PTGS2
BCL2A1
BCL2L2
Inflammation
IL1A
CD40LG
IL1B
CXCL2
IL2
PTGS2
IL6
ICAM1
IL8
VCAM1
CSF2
SELE
TNF
Angiogenesis
MMP1
MMP2
MMP3
MMP9
VEGFA
PTGS2
Proliferation
CCND1
VEGFA
UPR
HSF
Proteotoxic stress
ER
HSPA5
ERN1
EIF2AK3
ATF6
Proteotoxic stress
NFE2L2
EIF2A
HSF1
Gene transcription
XBP1
ATF4
ATF6
HSF1
Protein (re)folding, ERAD, UPR, apoptosis, amino acid metabolism
Protein (re)folding
Inflammation
HSPA5
DNAJB9
EDEM1
UBE2E1
PDIA6
XBP1
ATF6
ATF3
WARS
DNAJB9
BBC3
DNAJB11
BCL2L11
DNAJC3
DDIT3
ASNS
PPP1R15A
SULT1E1
TRIB3
NARS
HSPA5
HSP90B1
CALR
XBP1
ERP27
DDIT3
HSPA1A
HSPA5
HSPA6
HSPB1
HSPB2
HSP90AA1
DNAJB1
DNAJB4
DNAJC3
JUN
FOS
IL6
NFE2L2
MAPK8
MAPK11
MAPK13
MAPK12
MAPK14
KEAP1
OH
NFE2L2
KEAP1
ROS
NFE2L2
Gene transcription
NFE2L2
JUN
FOS
Multidrug resistance
ABCC2
ABCC3
ABCC4
ABCC6
ABCG2
Antioxidant synthesis/recycling
HMOX1
GCLC
GCLM
GSTP1
Neutralization of toxins
NQO1
NQO2
EPHX1
SRXN1
CES1
HIF-1
ROS / Hypoxia
EGLN1
HIF1AN
X
X
HIF1A
Gene transcription
HIF1A
ARNT
Apoptosis
TP53
BAK1
BAX
BID
BNIP3
BNIP3L
PMAIP1
Angiogenesis
VEGFA
PTGS2
EDN1
SERPINE1
NOS2
ANGPT1
ANGPT2
EPO
TGFA
TGFB3
Glycolysis
SLC2A1
SLC2A3
HK1
LDHA
PKM2
SLC16A1
PDHA1
PFKL
PGK1
Proliferation
VEGFA
TGFA
TGFB3
IGFBP1
IGFBP2
IGFBP3
Survival
BCL2A1
BCL2L1
MCL1
BIRC5
PTGS2
Legend
LC50
LC90
Log₂ fold-change
-1.0
0.0
1.0
No data found

Supplementary Figure S3. Cell type-dependent PDT-induced survival signaling. Transcriptomic response of HUVEC, RAW 264.7, SK-ChA-1, and A431 cells following ZPCL-PDT at the LC_{50} and LC_{90} concentrations. Gene expression data were mapped onto AP-1- (top), NF-κB- (middle left), UPR- (middle right), NFE2L2- (bottom left), and HIF-1α-mediated pathways (bottom right), irrespective of *P*-value. The color and intensity of the box indicates the direction and extent of the log_2 fold-change (versus the control group) for the indicated gene, respectively (legend lower left). Grey boxes signify probes that exhibited poor quality or were not included in the gene expression analysis. Each gene box, which may comprise multiple probes as indicated by horizontal splits, is vertically divided in two halves corresponding to the LC_{50} and LC_{90} groups (legend lower left). Abbreviations: ER, endoplasmic reticulum; ERAD, ER-associated degradation.

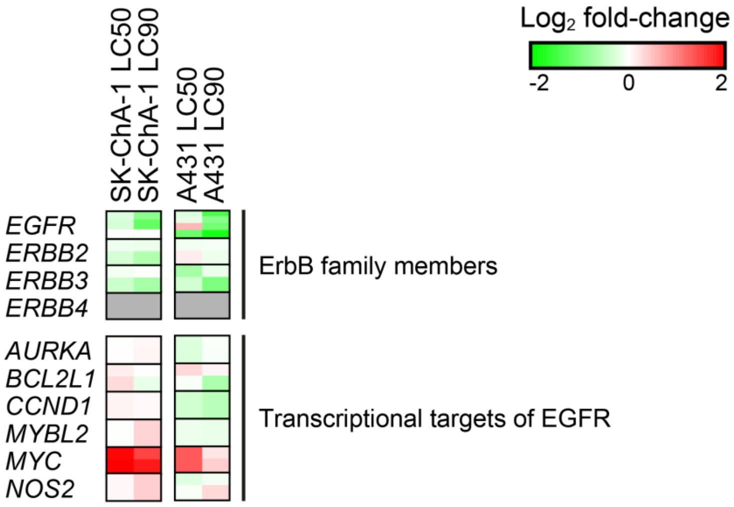

Supplementary Figure S4. Analysis of EGFR signaling after ZPCL-PDT. Gene expression is depicted as the log_2 fold-change (upper right corner) between the PDT-treated groups versus the control group (*n* = 3 per group). A gene may consist of multiple probes as indicated by horizontal splits. Grey boxes signify probes that exhibited poor quality or were not included in the gene expression analysis.

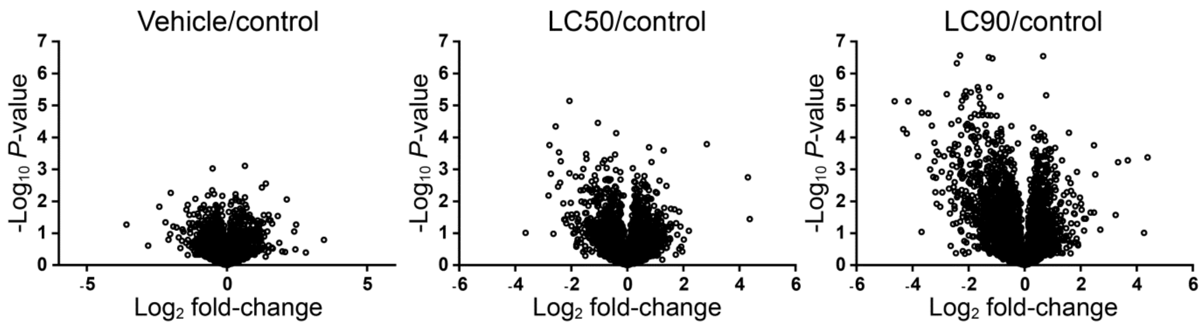

Supplementary Figure S5. Differentially expressed proteins as visualized by volcano plots. The group comparisons show $\log_2$ fold-changes of proteins and corresponding *P*-values observed in SK-ChA-1 cells subjected to ZPCL-PDT.

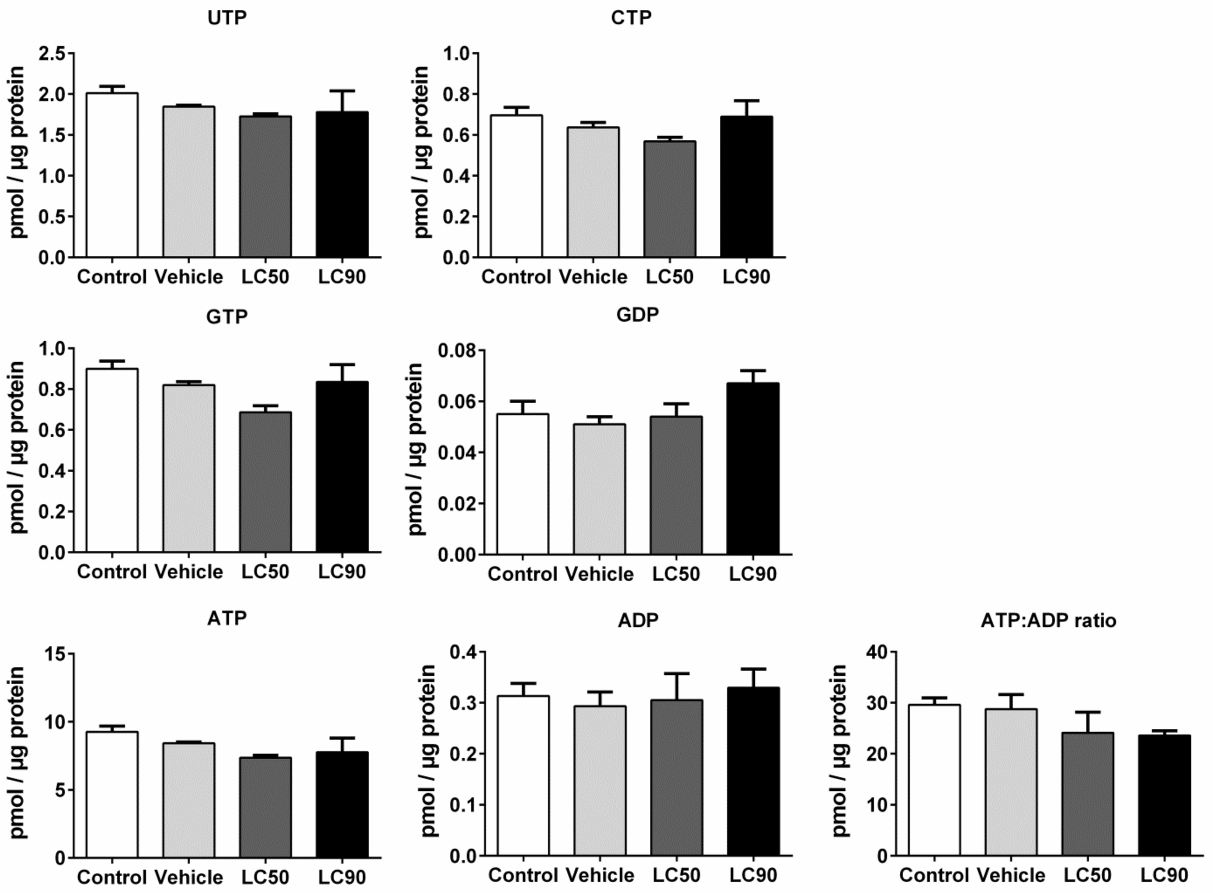

Supplementary Figure S6. Nucleotide analysis of SK-ChA-1 cells after ZPCL-PDT. Nucleotides were quantified using HPLC and depicted as pmol / µg protein (*n* = 3 per group).

A

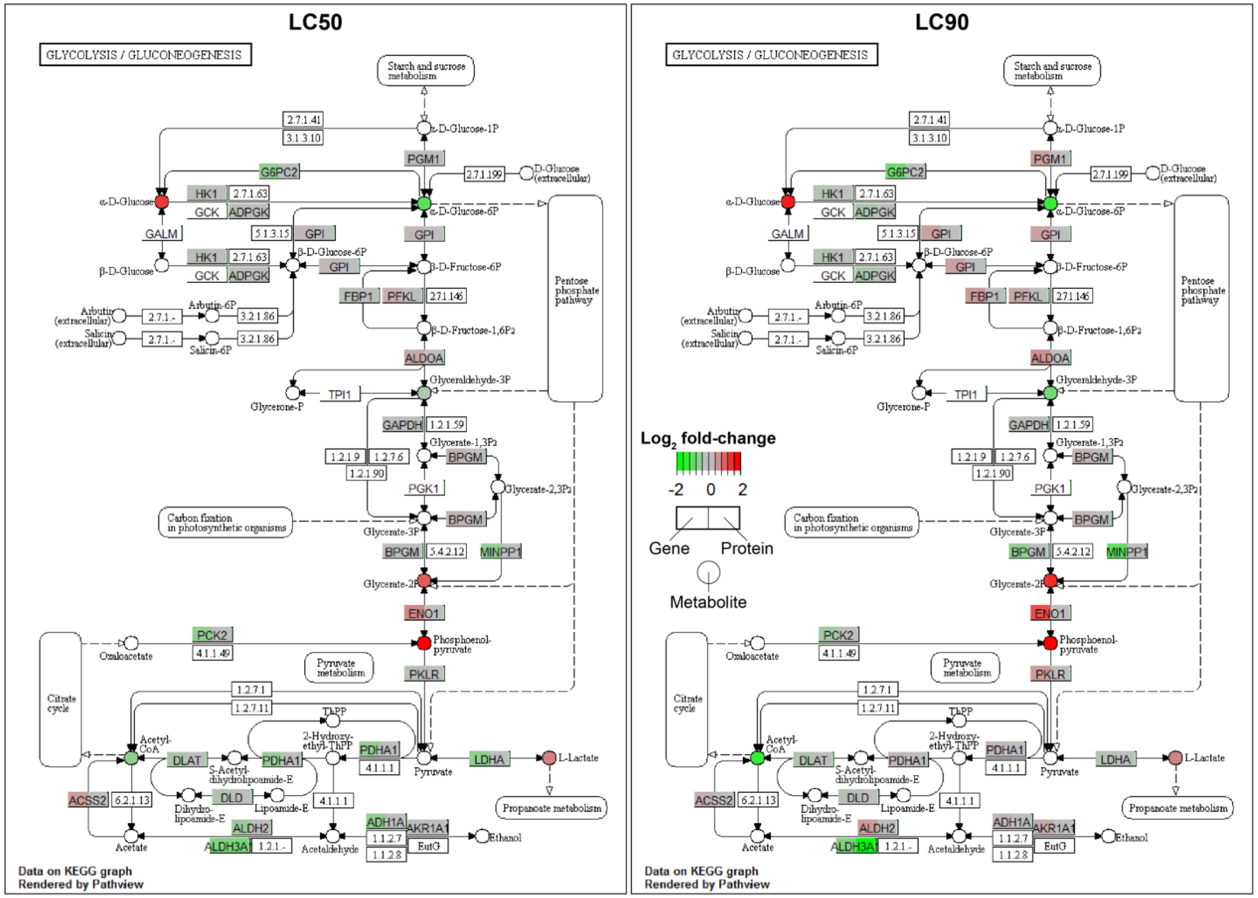
LC50
LC90
GLYCOLYSIS / GLUCONEOGENESIS
Starch and sucrose metabolism
α-D-Glucose-1P
PGM1
G6PC2
α-D-Glucose
α-D-Glucose-6P
D-Glucose (extracellular)
HK1
GCK
ADPGK
GALM
GPI
β-D-Glucose
β-D-Glucose-6P
β-D-Fructose-6P
FBP1
PFKL
β-D-Fructose-1,6P2
Arbutin (extracellular)
Arbutin-6P
Salicin (extracellular)
Salicin-6P
ALDOA
Glyceraldehyde-3P
TPI1
Glycerone-P
Pentose phosphate pathway
GAPDH
Glycerate-1,3P2
BPGM
Glycerate-2,3P2
PGK1
Carbon fixation in photosynthetic organisms
Glycerate-3P
MINPP1
Glycerate-2P
ENO1
PCK2
Oxaloacetate
Phosphoenol-pyruvate
Pyruvate metabolism
PKLR
Citrate cycle
Acetyl-CoA
DLAT
S-Acetyl-dihydrolipoamide-E
2-Hydroxy-ethyl-ThPP
ThPP
PDHA1
Pyruvate
LDHA
L-Lactate
Propanoate metabolism
ACSS2
Dihydro-lipoamide-E
DLD
Lipoamide-E
ALDH2
ALDH3A1
ADH1A
AKR1A1
EtoH
Ethanol
Acetate
Acetaldehyde
Log2 fold-change
-2
0
2
Gene
Protein
Metabolite
Data on KEGG graph
Rendered by Pathview

B

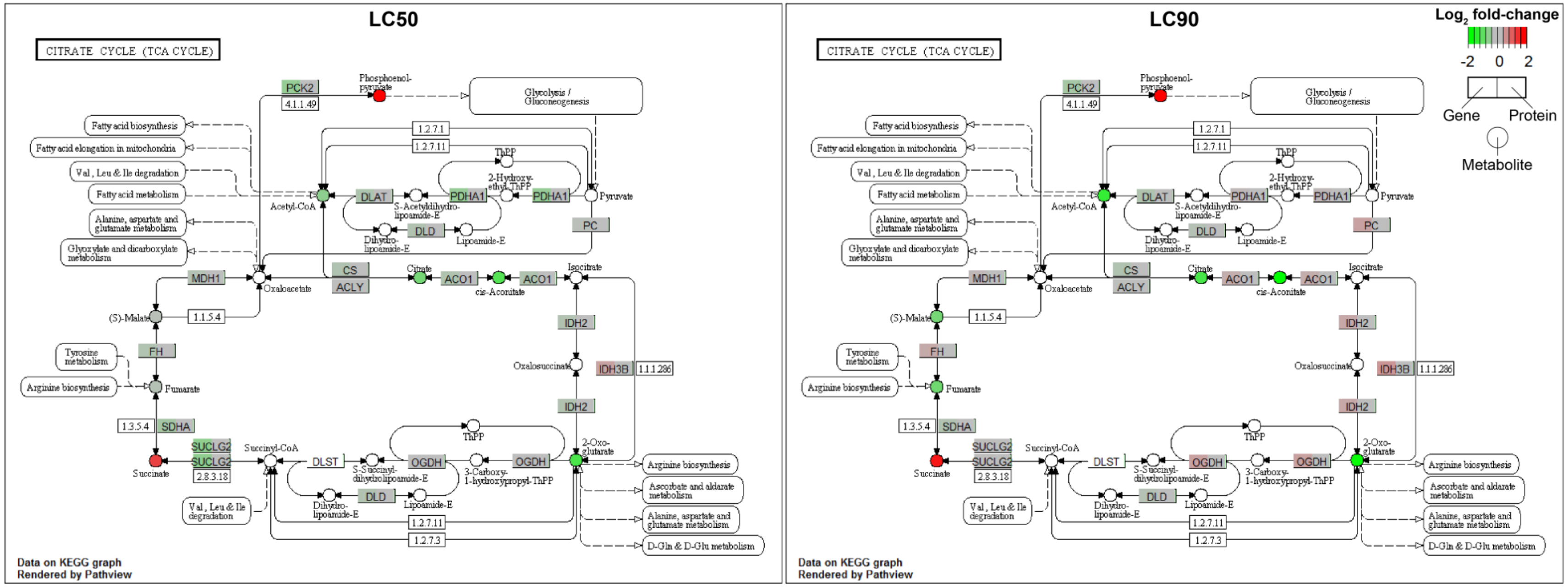
LC50
LC90
CITRATE CYCLE (TCA CYCLE)
Log2 fold-change
-2
0
2
Gene
Protein
Metabolite
Data on KEGG graph
Rendered by Pathview

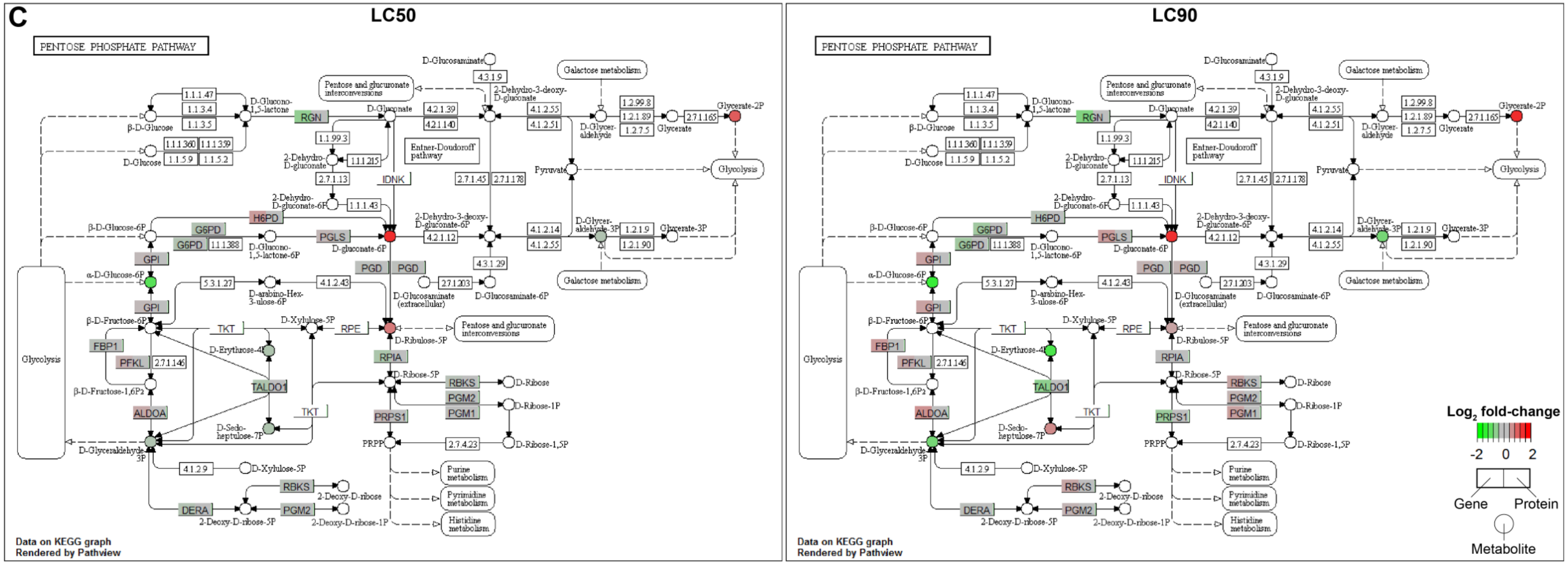

Supplementary Figure S7. Integration of transcriptomics, proteomics, and metabolomics data of SK-ChA-1 cells after ZPCL-PDT. Data was integrated for (A) the glycolysis/gluconeogenesis pathway, (B) tricarboxylic acid (TCA) cycle, and (C) the pentose phosphate pathway. Metabolites are indicated in circles and genes (left half of the square) and proteins (right half of the square) are indicated in squares. The left part of each pathway corresponds to the LC_{50} group, whereas the right part of each pathway corresponds to the LC_{90} group. Expression is depicted as the $\log_2$ fold-change between the treated groups versus the control group, where green and red represent down- and upregulation, respectively.
